# Effects of biomechanical and biochemical stimuli on angio- and vasculogenesis in a complex microvasculature-on-chip

**DOI:** 10.1016/j.isci.2023.106198

**Published:** 2023-02-13

**Authors:** Dario Ferrari, Arunima Sengupta, Lyong Heo, Laszlo Pethö, Johann Michler, Thomas Geiser, Vinicio A. de Jesus Perez, Wolfgang M. Kuebler, Soheila Zeinali, Olivier T. Guenat

**Affiliations:** 1 Organs-on-chip Technologies Laboratory, ARTORG Center, University of Bern, Bern, Switzerland; 2 Stanford Center for Genomics and Personalized Medicine, Palo Alto, CA, USA; 3 Empa, Swiss Federal Laboratories for Materials Science and Technology, Laboratory for Mechanics of Materials and Nanostructures, Thun, Switzerland; 4 Department of Pulmonary Medicine, Inselspital, University Hospital of Bern, Bern, Switzerland; 5 Department for BioMedical Research, University of Bern, Bern, Switzerland; 6 Division of Pulmonary, Allergy, and Critical Care Medicine, Stanford University Medical Center, Stanford, CA, USA; 7 Institute of Physiology, Charité - Universitätsmedizin Berlin, Berlin, Germany; 8 Department of General Thoracic Surgery, Inselspital, University Hospital of Bern, Bern, Switzerland

**Keywords:** Vascular anatomy, Biotechnology, Transcriptomics

## Abstract

The endothelium of blood vessels is a vital organ that reacts differently to subtle changes in stiffness and mechanical forces exerted on its environment (extracellular matrix (ECM)). Upon alteration of these biomechanical cues, endothelial cells initiate signaling pathways that govern vascular remodeling. The emerging organs-on-chip technologies allow the mimicking of complex microvasculature networks, identifying the combined or singular effects of these biomechanical or biochemical stimuli. Here, we present a microvasculature-on-chip model to investigate the singular effect of ECM stiffness and mechanical cyclic stretch on vascular development. Following two different approaches for vascular growth, the effect of ECM stiffness on sprouting angiogenesis and the effect of cyclic stretch on endothelial vasculogenesis are studied. Our results indicate that ECM hydrogel stiffness controls the size of the patterned vasculature and the density of sprouting angiogenesis. RNA sequencing shows that the cellular response to stretching is characterized by the upregulation of certain genes such as ANGPTL4+5, PDE1A, and PLEC.

## Highlights

• An open OOC device with two parallel channels and three-dimensional stretch is shown
• Different mechanical stimuli (cyclic stretch and stiffness) are applied on endothelial cells
• Sprouting angiogenesis and vasculogenesis models are investigated
• Next-generation sequencing of the patterned vessels was performed

## Graphical abstract

**Figure:**
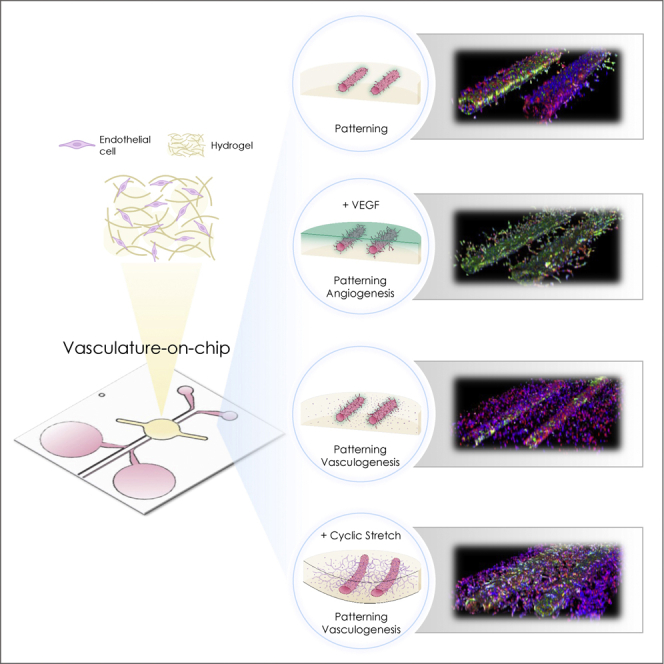

## Introduction

The human body vasculature system is hierarchical. Different network parts vary wildly in vessel morphology and dimensions, response to mechanical forces (e.g., shear stress, mechanical stretch, and ECM stiffness). Vasculature network formation is controlled by the dynamic cellular microenvironment, which modulates and triggers heterogeneous cell behaviors. Furthermore, interactions between endothelial cells and their microenvironment regulate oxygen, nutrient, and waste transport,^1^ as well as cellular and tissue functions.^2^ Any significant changes in these cues may affect the response of endothelial cells. For instance, pathological idiopathic pulmonary fibrosis and acute respiratory distress syndrome induce non-homogeneous lung remodeling, resulting in considerable vascular heterogeneity.^3^^,^^4^ However, much remains to be discovered about the mechanistic basis of vascular growth and remodeling affected by the cellular microenvironment in both normal and pathological conditions.

The creation of blood vessels can be separated into two major processes: angiogenesis and vasculogenesis. Angiogenesis describes the formation of new blood vessels from existing ones and includes sprouting and intussusceptive angiogenesis. During sprouting angiogenesis, new vessels branch from existing vessel, whereas intussusceptive angiogenesis describes the splitting of existing blood vessels. Vasculogenesis describes the *de novo* formation of new blood vessels and occurs mainly during the embryonic stage and during wound healing in adults.^5^^,^^6^

The creation of functional artificial vessels using endothelial cells is one of the significant successes of organs-on-chip (OOC) technology.^7^ Two primary methods have been reported to create such vessels: substrate templating with subsequent cell seeding (patterning) or exploiting the innate self-assembly capacity of endothelial cells (EC). These techniques have been widely used and combined, enabling the recapitulation of complex biological functions.^8^^,^^9^^,^^10^ In addition, several groups reported the formation of perfusable lumen structures through ECM hydrogels that mimic tubular-shaped vessels' *in vivo* structure and function. For example, tiny rods embedded in collagen hydrogel have been used to create tubes with a confluent layer of endothelial cells.^11^^,^^12^ The technique was further developed to combine channel patterning with cell seeding. Coated gold rods loaded with GFP-HUVEC cells were immersed in a gelatin solution, followed by electrochemical cell transfer.^13^^,^^14^ Although elegant, the latter technique requires to culture cells on a rod prior to transferring them in the hydrogel. Viscous finger patterning technique is another technique used to create patterned lumens of various dimensions coated with endothelial cells.^15^ Unlike the viscous finger patterning method, the rod technique is more delicate, but can be used to make parallel vessels. Nguyen et al. produced 400 μm-wide channels with an interchannel distance of 1 mm, one of which was lined with endothelial cells, whereas the second contained angiogenic factors. This experimental setup allowed the comparison of the effects of different angiogenic cocktails.^12^ OOC devices have been used to expose 3D vessels to different types of mechanical stimuli; fluid shear stresses influence sprouting, EC genetic differentiation, vessel permeability, and differential EC gene and protein expression. In contrast, interstitial flow affected cell migration, capillary formation, sprout lengths, and growth factor gradients.^16^^,^^17^^,^^18^ Mechanical strain is also known to affect the endothelial response,^19^ although it has so far mostly been investigated using uni- or biaxial strain on two-dimensional endothelial cell monolayers. The effects of mechanical strain on 3D endothelialized lumens are only starting to be investigated.^20^^,^^21^ Equilateral strain can be transmitted on 3D hydrogel constructs loaded with human cells, mimicking the cyclic stretch the alveolar sac capillaries are exposed to during the breathing motion and through intraluminal pressure.^22^ Zeinali et al. used equilateral stretching of a single channel lined with ECs inside a fibrin hydrogel to demonstrate the effects of different stretch magnitudes and found that cyclic stretch increases the cell length and surface area.^23^ Recently, Shimizu et al.^20^ presented a stretchable microfluidic culture system with two perfusable parallel channels. Using transglutaminase-crosslinked gelatin as a substrate, the channels were produced through sacrificial molding. Using this system, they demonstrated that a simultaneous application of shear stress and planar stretching on endothelial cell alignment are additive.^20^ The effect of ECM stiffness—another mechanical stimulus of the cellular microenvironment—was reported to regulate vasculogenesis in hydrogel^24^ and in a microfluidic cell culture system.^25^

In contrast to previous studies, we present here for the first time the effects of mechanical stimuli (3D cyclic mechanical stress and ECM stiffness) on sprouting angiogenesis and on endothelial vasculogenesis using a complex open-top microvasculature-on-chip. An optimized microfabrication technique associated with vasculature patterning is presented, allowing for the reproducible creation of two parallel vessels of approximately 150–200 μm in size and 500 μm apart in a hydrogel matrix. Using two fibrin hydrogel concentrations, we investigate the effect of slight variations in ECM stiffness on angiogenesis sprouting from patterned vessels. The open-top microfluidic enables the topical application of nutrients and/or compounds. We also examined the effects of equilateral 3D cyclic stretch (abbreviated “stretch”) on endothelial vasculogenesis around mature patterned vessels. Furthermore, we performed next-generation sequencing to uncover differences in gene expression between static and cyclic stretch conditions. This platform not only enables a better understanding of the sprouting angiogenesis and endothelial vasculogenesis processes upon biomechanical and biochemical stimuli but also develops complex and versatile microvasculature-on-chip models by combining the approaches of patterning and self-assembly *in vitro*.

## Results

### Creating two parallel endothelialized vessels

**Figure 1:**
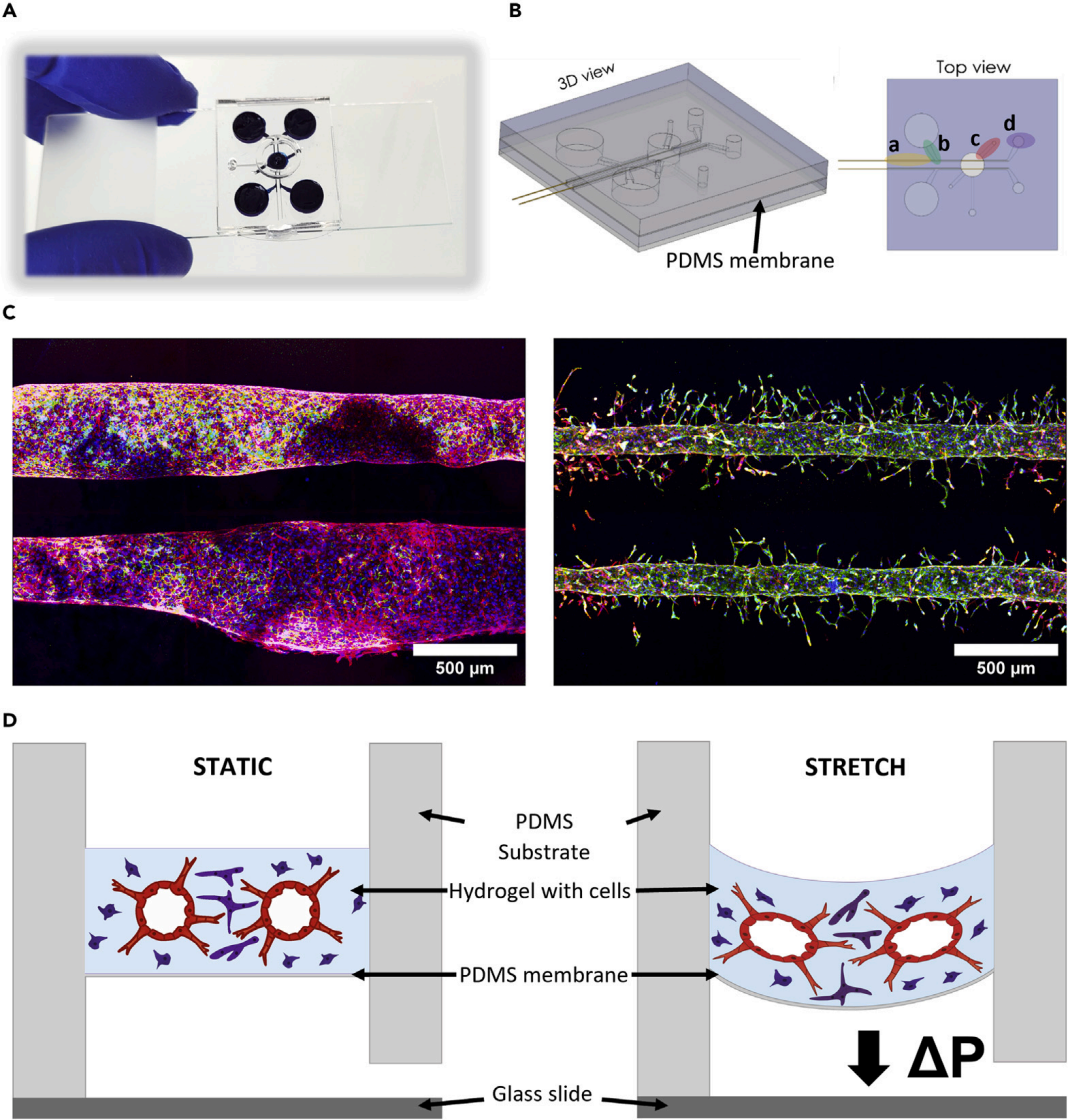
Microfluidic platform to re-create 3D microvasculatures *in vitro* (A) Photograph of the complete microfluidic chip made from PDMS, residing on a microscopy glass slide. (B) 3D schematic illustration of the microfluidic chip. Top view: a) rod removal guides, b) pipette tip connection, c) gel seeding port, d) second medium well. (C) Two parallel microvessels generated from different design iterations, without (left) and with (right) removal guides). (blue: Hoechst, green: Pecam-1, red: F-Actin, Day: 8) (D) Cross-sectional schematic of the deflection process during cyclic stretch.

A combination of two techniques, vessel patterning and endothelial cell self-assembly, was used to create a vascular network. The initial design of the chip used two large reservoirs at the end of each lumen (Figure S1); however, rod removal proved to be a delicate operation, resulting in inhomogeneous diameter of the vessels (Figure 1C). Improving the design (Figure 1A) led to greater vessel homogeneity (Figure 1C). Modifications included the introduction of rod removal guides, optimized pipette tip connections from the medium wells to the channels, elongated gel seeding ports to minimize bubble entrapment, and a variable second medium well size for optional connection to tubing (Figure 1B). Figure 1B shows the schematic of the final design, with a cross-sectional and top view. The rods are longer than the channels of the chip, such that they can be removed later on with tweezers. Here, the rod removal guides proved to be crucial, since friction forces seemed to prevent distortion of the rods during removal. Subsequent endothelial cell seeding into the parallel lumens from their reservoirs resulted in the homogeneous coating of the vessel walls (Figure S2). Homogeneous vessels formation was tested with two hydrogels (Figure 3). The fibrin hydrogel layer lies on a 100 μm thin polydimethylsiloxane (PDMS) membrane spanning a cavity (Figure 1B). When exposed to negative cyclic pressure, the membrane deflects the vessel-hydrogel construct, affecting the endothelial cells. The open-access design and transparent PDMS allow for real-time observation of cells cultured on the chip, as well as independently perfusable vessels (Figures S3 and S4).

### Approaches for microvasculature growth around patterned vessels

**Figure 2:**
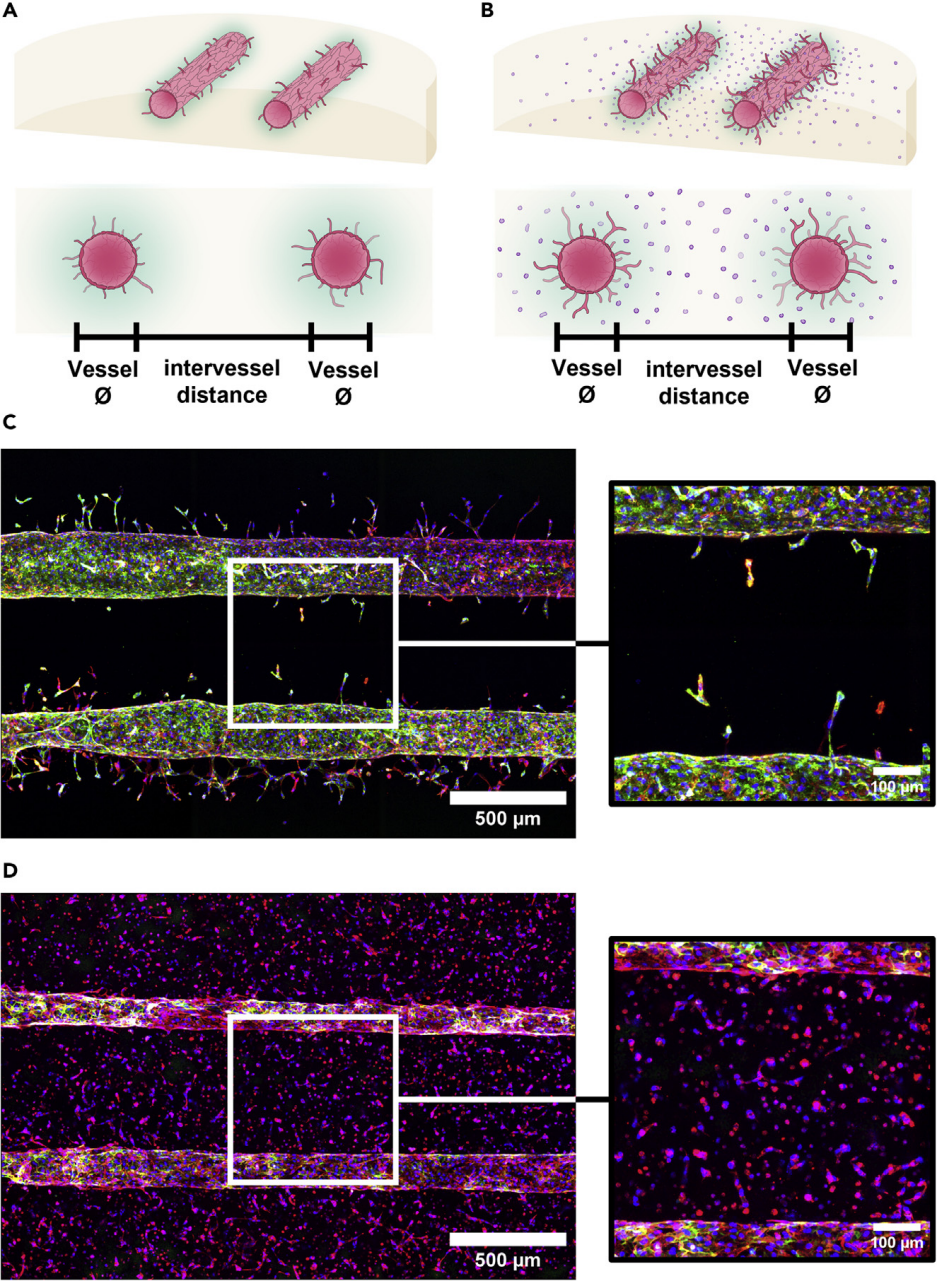
Establishment of the microvasculature formation and development (A+B) Schematic of the patterning of two parallel microvessels into fibrin hydrogel to study sprouting angiogenesis (A) and vasculogenesis (B). (C) Exemplary z-projected fluorescence microscope images of two parallel microvasculatures and a close-up view of the region between the vessels. (blue: Hoechst, green: Pecam-1, red: F-Actin, Day: 8) (D) Exemplary z-projected fluorescence microscope images of two parallel microvasculatures surrounded by endothelial cells within the hydrogel matrix and a close-up view of the region between the vessels. (blue: Hoechst, green: Pecam-1, red: F-Actin, Day: 5).

**Figure 3:**
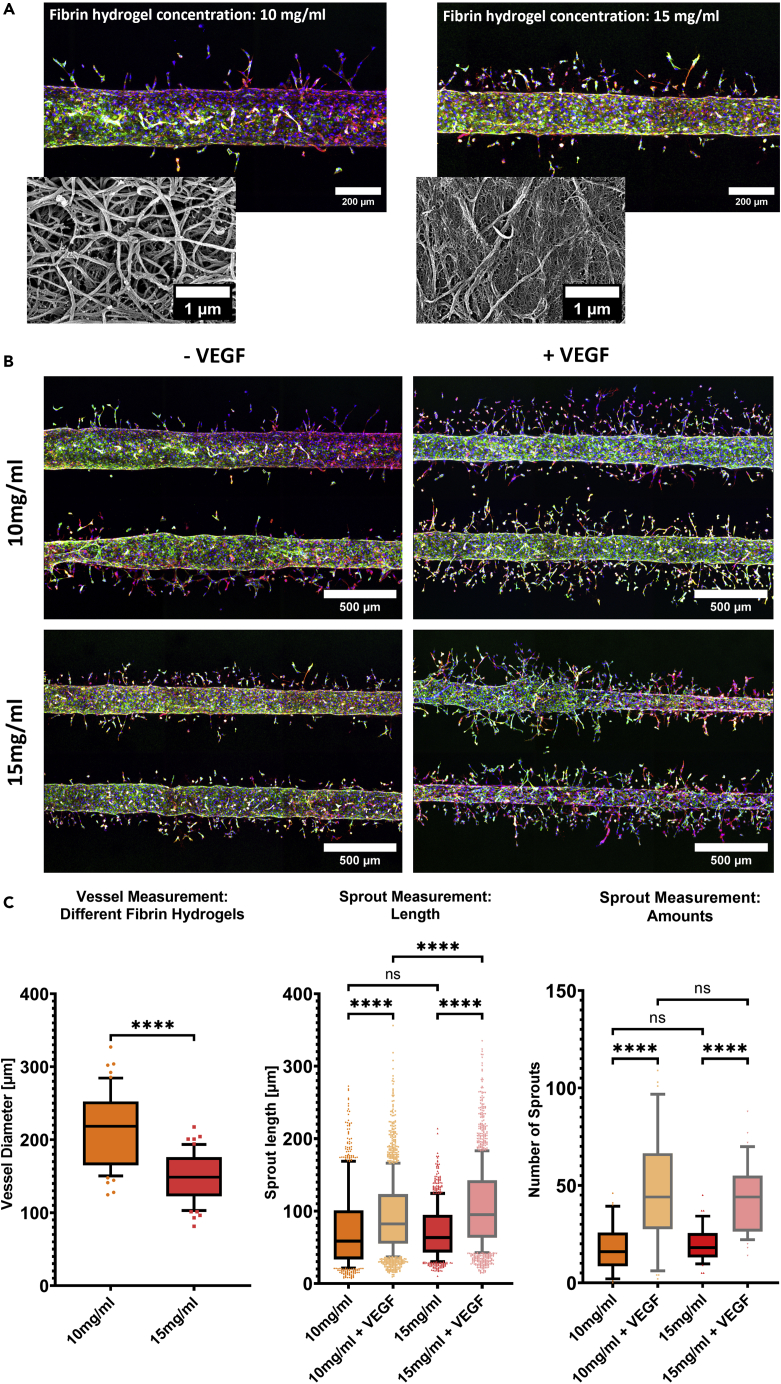
Effect of different hydrogel stiffness and VEGF on vascular size and angiogenic sprouting (A) Exemplary z-projected fluorescence microscope images of a vessel patterned in different fibrin hydrogel concentrations, with the smaller pictures depicting SEM scans at a 30′000× magnification (blue: Hoechst, green: Pecam-1, red: F-Actin, Day: 8). (B) Effect of VEGF on angiogenesis in different fibrin hydrogel concentrations. Overview of the different conditions with exemplary z-projected fluorescence microscope images (blue: Hoechst, green: Pecam-1, red: F-Actin, Day: 8). (C) From left to right: Vessel diameter measurements in different fibrin hydrogel concentrations (whiskers: 10–90 percentile, unpaired t-test, p value <0.0001: ∗∗∗∗; p value <0.001: ∗∗∗; p value <0.01: ∗∗; p value <0.05: ∗, p value >0.05: ns, N = 3, total n = 9/condition), sprout length, and amount measurements of vessels in different fibrin hydrogel concentrations, with and without the addition of VEGF (whiskers: 10–90 percentile, ordinary one-way ANOVA, p value <0.0001: ∗∗∗∗; p value <0.001: ∗∗∗; p value <0.01: ∗∗; p value <0.05: ∗, p value >0.05: ns, N = 3, total n = 9/condition).

**Figure 4:**
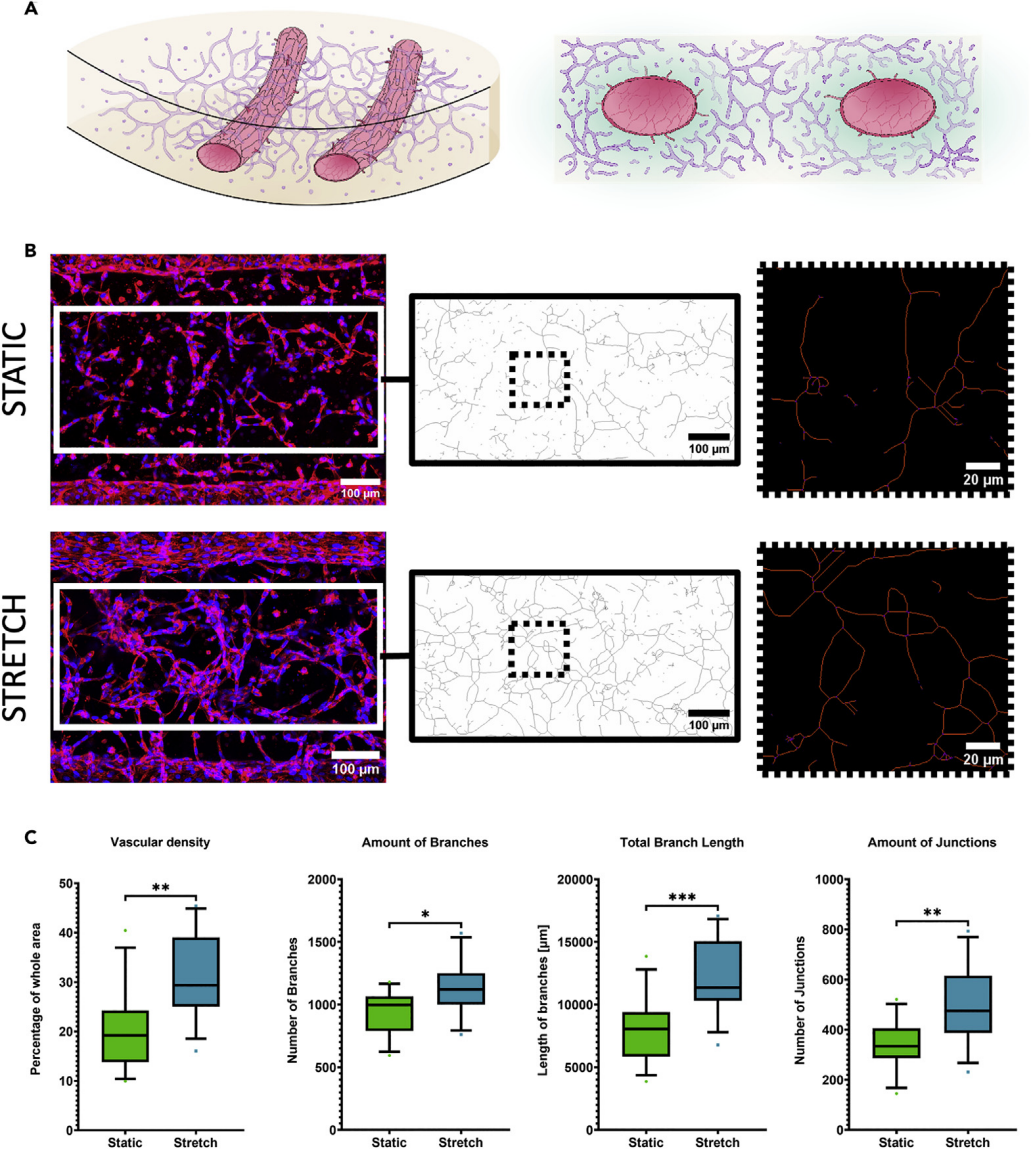
Effect of mechanical cyclic stretch on vasculogenesis (A) Schematic illustration of the model. Left: 3D drawing of the deflected fibrin hydrogel with vessels. Right: Cross-sectional view. (B) From left to right: Examples of the intervessel area of z-projected fluorescence microscope images from the vasculogenesis model under static (top row) and stretch (bottom row) conditions, skeletonized intervessel space after the segmentation, and close-up view after quantification. (blue: Hoechst, red: F-actin, Day: 5) (C) From left to right: Vascular density (part of the total area covered by vascular network based on segmented images), amount of branches, total branch length, and amount of junctions (unpaired t-test, p value <0.0001: ∗∗∗∗; p value <0.001: ∗∗∗; p value <0.01: ∗∗; p value <0.05: ∗, p value >0.05: ns, N = 3, total n = 12/condition).

We used two approaches to model different aspects of vessel developments (Figure 2). The first approach induces sprouting angiogenesis from the patterned microvasculatures with vascular endothelial growth factor (VEGF) treatment (Figures 2A, 2C, and 3). The second approach allows for vascular self-assembly around the patterned vasculatures with the endothelial cells embedded within the hydrogel (Figures 2B, 2D, and 4).

### The interplay of hydrogel stiffness and VEGF treatment in sprouting angiogenesis

To understand the contribution of hydrogel stiffness in sprouting angiogenesis, two fibrin hydrogel concentrations of 15 and 10 mg/mL were used. Scanning electron microscopy revealed differences in porosity and density of the hydrogel matrices (Figures 3 and S5). We compared vessel fabrication reproducibility and the influence of VEGF on sprout formation between the two hydrogel concentrations. The difference in fibrin hydrogel concentration impacted the final vessel diameter. The vessels created in the softer fibrin gel resulted in a mean diameter of about 220 μm (the patterning rod is 100 μm-in-diameter), whereas the higher concentrated fibrin solution (15 mg/mL) led to the formation of smaller vessels (mean 150 μm) (Figures 3A and 3C). Sprouts measurements under four different conditions were conducted, namely 10 or 15 mg/mL fibrin hydrogel solutions with or without VEGF addition (Figure 3B). Sprouts were significantly longer and more numerous upon exposure to VEGF in both hydrogels. The sprout lengths had a mean length of about 90 μm in the softer hydrogel and of about 100 μm in the stiffer one. Without VEGF, sprouts in both hydrogels had similar lengths with a mean of about 70 μm. The amount of sprouts was also similar in both hydrogels (about 20 without VEGF), with a significant increase (about 45 with VEGF) upon exposure to VEGF (Figure 3C). Furthermore, the amount and length of sprouts differed whether they were lateral or medial to the vessel, an effect that was less pronounced under the 15 mg/mL condition without VEGF (Figure S6). The sprouts located between the two vessels (medially) were less numerous and shorter than those located laterally (Figure S6). This can be explained by the higher amount of nutrients and VEGF on the lateral sides of the vessel compared with the amount available between the two vessels. A prolonged culture time for the untreated 15 mg/mL condition showed maximum sprouts' length at day 8 (Figure S7).

### Effect of cyclic stretch on vasculogenesis *in vitro*

The previous results indicated better vessel creation with the higher concentrated fibrin hydrogel, so subsequent experiments used this concentration. A model with complementary vasculogenesis was developed by suspending additional endothelial cells into the hydrogel precursor solution (Figure 4A). Here, the effect of cyclic stretch was investigated on three different parts: the intervessel network density, the morphology of the (patterned) vessel wall lining cells, and the shape of the whole (patterned) vessel. For the morphology of the vessel wall lining cells and the shape of the whole vessel, additional spatial comparisons were conducted. The ratio of the vascularized area to total area revealed that cyclic stretch increased vascular density (mean fold change: 1.5). Furthermore, the amount of branches, the total branch length, and the amount of junctions were increased under stretch condition (Figure 4C). Naturally, the fixed boundary of the hydrogel cell construct experienced the least amount of strain. Measurements were taken from z-projected immunofluorescence images of the intervessel area of the same size, located in the middle of the chip. Automated subsequent segmentation (Figure S8) and network area measurements revealed a denser network under the influence of cyclic stretch. The skeletonization depicted in Figure 4B was used to analyze interconnectivity of the vascular network.

### Effect of the 3D cyclic stretch on cell and vessel morphology of the patterned vessels

**Figure 5:**
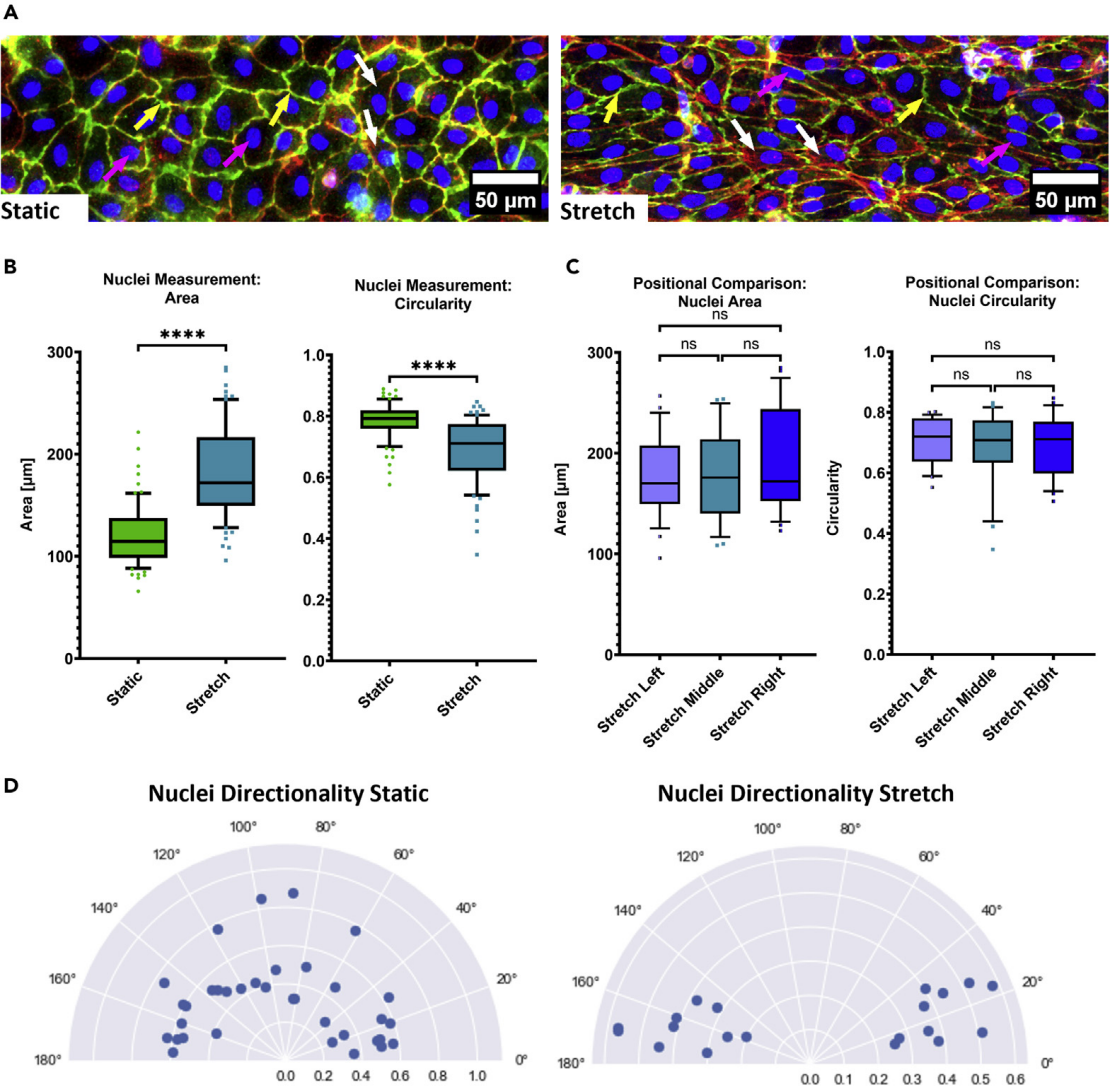
Effect of the mechanical cyclic stretch on the morphology of the vessel wall lining cells (A) Exemplary z-projected fluorescence microscope close-up images of the vessel wall. Yellow arrows indicate Pecam-1 expression (plasma membrane), whereas pink arrows indicate the shape and orientation of the nuclei, and white arrows F-Actin stained fibers (blue: Hoechst, green: Pecam-1, red: F-Actin, Day: 5). (B) Comparison of nuclei morphology, namely area and circularity between static and stretch condition (whiskers: 10–90 percentile, unpaired t-test, p value <0.0001: ∗∗∗∗; p value <0.001: ∗∗∗; p value <0.01: ∗∗; p value <0.05: ∗, p value >0.05: ns, N = 3, total n = 12/condition). (C) Positional comparison of nuclei morphology under stretch condition (whiskers: 10–90 percentile, ordinary one-way ANOVA, p value <0.0001: ∗∗∗∗; p value <0.001: ∗∗∗; p value <0.01: ∗∗; p value <0.05: ∗, p value >0.05: ns, N = 3, total n = 12) (D) Comparison of nuclei orientation, where 0° or 180° is the direction of the main vessel, and 90° would be perpendicular to it. Plotted is the main direction of each analyzed segment with the relative amount on the radial axis. Only segments passing a Gaussian curve fit of the histogram with a goodness of ≥0.6 were taken (N = 3, total n = 12/condition).

**Figure 6:**
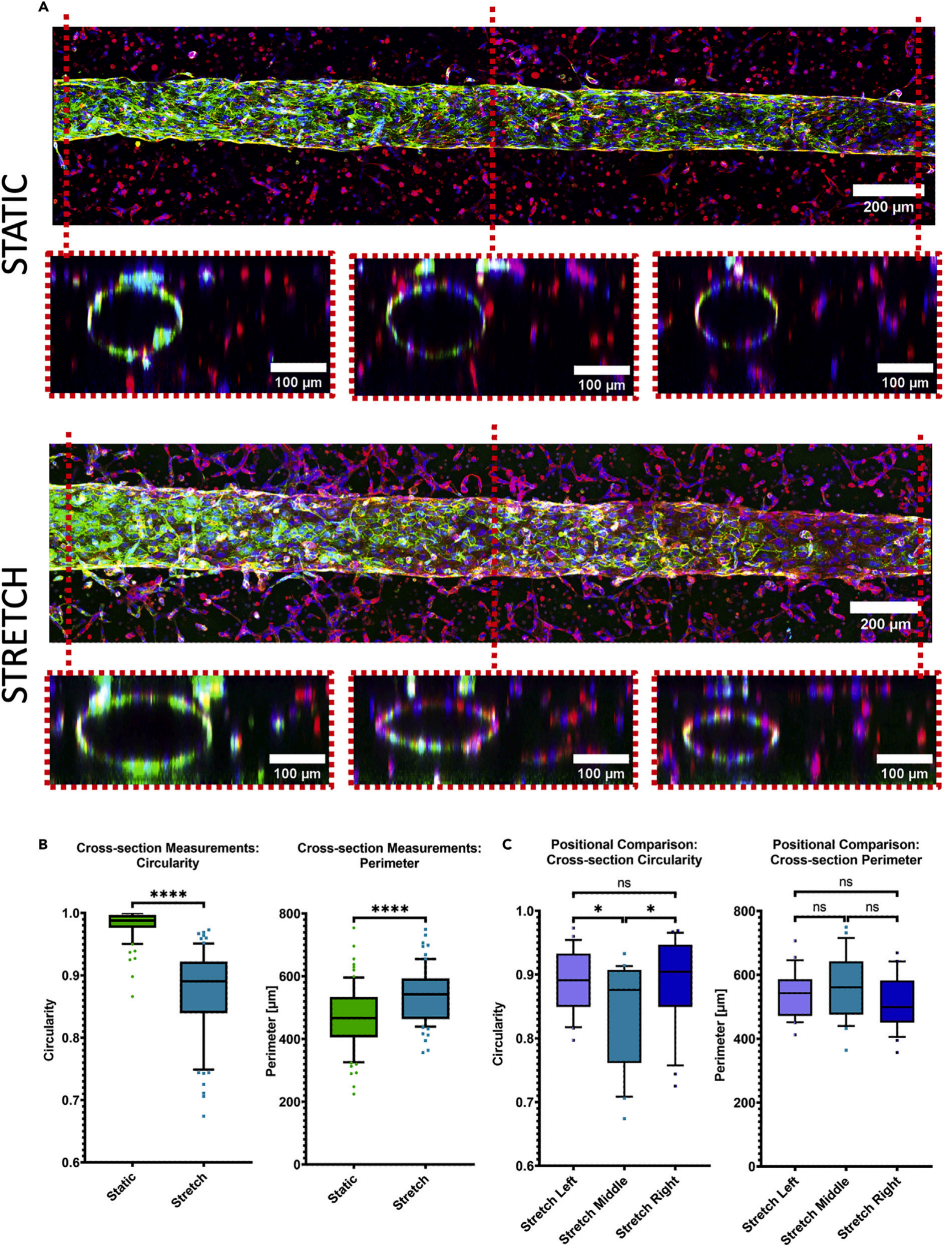
Effect of cyclic stretch on the morphology of the whole vessel (A) Exemplary z-projected fluorescence microscope images of one complete vessel with sliced cross-sections according to the position underneath, under static (top) and stretch (bottom) condition (blue: Hoechst, green: Pecam-1, red: F-Actin, Day: 5). (B) Comparison of the shape of the cross-sections regarding circularity of an overlaid ellipse and the size of its perimeter between the two conditions (unpaired t-test, p value <0.0001: ∗∗∗∗; p value <0.001: ∗∗∗; p value <0.01: ∗∗; p value <0.05: ∗, p value >0.05: ns, N = 3, total n = 12/condition). (C) Comparison of the cross-sections at different positions regarding circularity of an overlaid ellipse and the size of its perimeter under stretch condition (whiskers: 10–90 percentile, ordinary one-way ANOVA, p value <0.0001: ∗∗∗∗; p value <0.001: ∗∗∗; p value <0.01: ∗∗; p value <0.05: ∗, p value >0.05: ns, N = 3, total n = 12).

In the presented model, the effects of cyclic stretch can be measured at a cellular and vascular level. 3D cyclic stretch changes the morphology of the vessel wall lining cells (Figure 5) and also of the whole vessels (Figure 6).

Cells under cyclic stretch were more elongated, larger, and oriented in the same direction along the main vessel (Figure 5A). Measuring the shape of the nuclei revealed a larger area and a lower circularity under stretch condition (Figure 5B). Comparisons of the shape of the nuclei in dependency of their relative location in the main vessel revealed no significant differences under stretch (Figure 5C) or static (Figure S9B) condition. The directionality of the skeletonized nuclei also showed increased alignment in the direction of the main vessel under stretch condition (Figure 5D). These automated measurements confirmed initial manual measurements of the cells (Figure S9A).

The cross-section of vessels was more elliptical rather than being circular after stretch, quantified through the circularity measurement of an overlaid ellipse. Additionally, the cross-section perimeter was significantly increased (Figure 6B). Comparing these parameters in dependence of their relative position (Figure 6A) of the vessel—left end, middle part, and right end—revealed no difference under static condition (Figure S10), unlike the cross-section circularity under stretch (Figure 6C) condition.

### Impact of cyclic stretch on the transcriptome profile of cells

**Figure 7:**
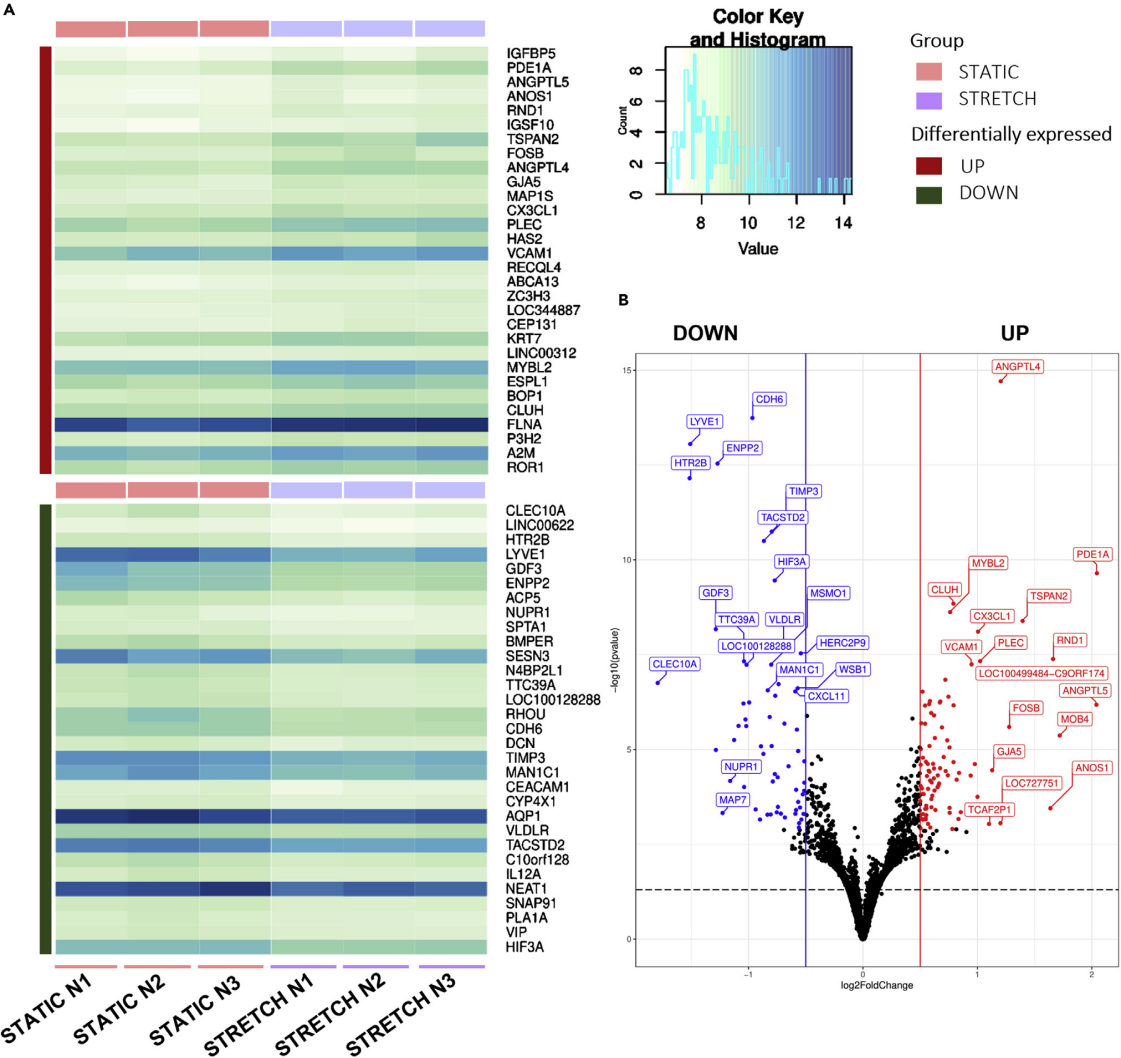
Molecular effects of cyclic stretch on endothelial cells lining the walls of the patterned vessel (A) Hierarchical clustering of differentially expressed genes as a heatmap, displaying the mean expression of 30 most up- (top section) and down (bottom section) regulated genes between static and stretch samples. Light green to dark blue refers to low to high relative expression levels of genes. (B) Volcano plot of the DEGs, with the threshold at a log_2_fold change (>0.5) (padj. < 0.05, N = 1, n = 3).

To get a better understanding of the gene expression differences resulting in the distinct morphologies under cyclic stretch and static, we performed RNA-sequencing to uncover differentially expressed genes (DEGs). Cells were collected from the patterned, large vessels of the vasculogenesis model, whereas the cells suspended inside the hydrogel were left out. The 30 most significant up- and downregulated genes are represented with heatmaps (Figure 7A), using respective color mapping for each sample. We focused on the DEGs in static cells compared to the stretched samples with cutoff criteria of log_2_ fold change higher than 1.0 and a significant adjusted p value <0.05 (Figure 7B and Table S1).

### Gene Ontology (GO) and Kyoto Encyclopedia of Genes and Genomes (KEGG) analyses

**Figure 8:**
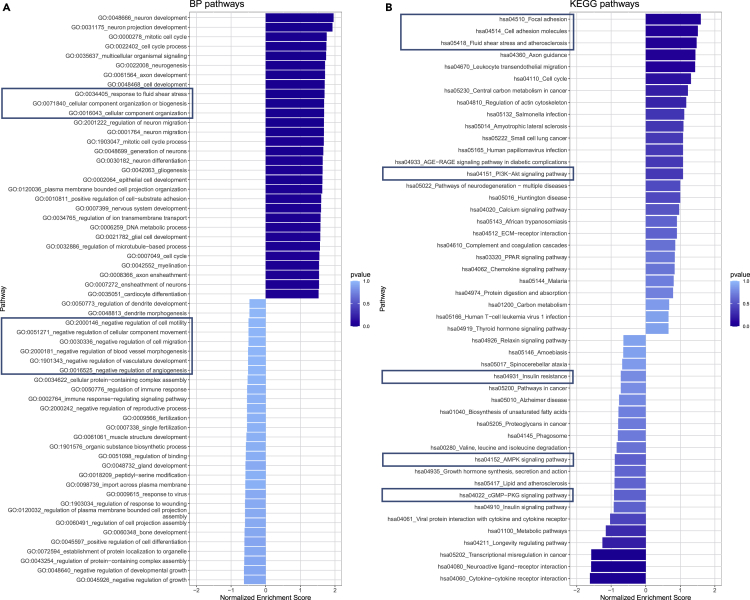
Biological pathway analysis of the NGS data Biological pathway analysis of the NGS data: (A) GO:BP pathways. (B) KEGG pathways. Pathways of special interest are highlighted with frames.

Pathway analysis was performed to identify essential pathways regulated by the DEGs identified above. In cells exposed to cyclic stretch, several well-established signaling pathways were induced, including response to fluid shear stress and cellular component organization in the GO:BP pathway (Figure 8A). GO:MF pathways further revealed upregulated GTPase binding, cytoskeletal motor activity, and microtubule motor activity, while GO:CC pathways revealed upregulated extracellular matrix, cytoskeleton, and polymeric cytoskeletal fibers under stretch (Figure S12). KEGG pathways exhibited upregulated focal adhesion, cell adhesion molecules, fluid shear stress and atherosclerosis, and P13-Akt signaling to be upregulated under stretch (Figure 8B).

## Discussion

This study enabled to investigate the effect of ECM hydrogel stiffness and 3D mechanical cyclic stretch on vascular development using a microvasculature-on-chip model. Two approaches for vascular growth are investigated: sprouting angiogenesis from pre-existing patterned vessels within hydrogel matrix upon treatment with VEGF and *de novo* vasculogenesis from endothelial self-assembly around the patterned vessels. Considering the importance of biophysical aspects of the endothelium microenvironment, the effect of ECM hydrogel stiffness on sprouting angiogenesis is studied. Regarding biomechanical forces from parenchyma on the vessels, the effect of cyclic stretch on endothelial vasculogenesis is investigated. Our results indicate that ECM stiffness controls the size of the patterned microvasculature and the density of the sprouting angiogenesis from this patterned vessel. Concentrated fibrin hydrogel matrix (15 mg/mL) results in creation of smaller patterned vessels and influences the length of the sprouts when compared to 10 mg/mL fibrin hydrogel. Cyclic stretch seems to increase endothelial vasculogenesis as measured between the two patterned vessels. The density of the microvasculature network formed by vasculogenesis significantly increases after 24 h of 3D cyclic stretch in comparison to static condition. RNA sequencing was applied to obtain a comprehensive understanding of mechanical cyclic stretch-regulated genes and pathways in endothelial cells forming patterned vessels. Cellular response to stretch was represented by top upregulated genes such as ANGPTL4+5, RND1, PLEC, and PDE1A, suggesting restructuring and proliferative effects of stretch.

### Vessel patterning in ECM hydrogel

This paper describes a method for vessel formation using a microfluidic chamber with a hydrogel. We found that the resulting size of the lumen was larger than the rod diameter (100 μm) used to pattern them. This may result from friction forces occurring during rod removal. In addition, shear stress forces induced during the introduction of cells and the addition of culture medium might dilate the patterned vessels. Hydrogel concentration also plays an important role, shown through an average 15% increase in the vessels’ diameter in the softer hydrogel compared to the stiffer one. The stiffness difference between the tested hydrogels corresponds to about 0.5–1 kPa.^26^^,^^27^ In addition, cells decompose and produce ECM over the culture time, although this likely plays only a minor role in the observed effect. Nevertheless, we successfully calibrated and validated a reliable method of engineering parallel vessels with a diameter of around 150–200 μm and a horizontal distance of about 500 μm. Similar changes of channel parameters due to cell culture and gelation conditions were observed using collagen type 1 as a hydrogel.^11^ The possibility to create parallel vessels with a high geometrical reproducibility is an important step forward to create even more complex vasculature systems, for instance with vessels of different diameters.

### Sprouting angiogenesis in different fibrin hydrogel concentrations

The open-chamber design of the platform allows for adding VEGF to the patterned vessels to induce sprouting angiogenesis. Stiffer fibrin hydrogel in combination with VEGF resulted in a significantly stronger effect with regards to the length of sprouts. Manual measurements and sampling numbers probably contributed to the observation of several randomized effects, such as the non-significant difference in sprouts length between the 10 and 15 mg/mL fibrin hydrogel without VEGF. This result, however, does not detract from the developed VEGF-treated microfluidic device as a proof-of-concept for drug testing. Previous studies have investigated the effect of VEGF on inducing sprouting angiogenesis under different conditions. For example, Song and Munn used a microfluidic device with a VEGF gradient and found that VEGF-stimulated HUVEC-GFP cells migrated into the bulk of the ECM space and showed a distinct morphology.^28^

### Effect of the 3D cyclic stretch on cell and vessel morphology of the patterned vessels

The design of our microvasculature-on-chip platform enabled us to generate two parallel microvasculatures created by patterning within fibrin hydrogel, incorporating a population of endothelial cells. Starting from seeding day, *de novo* vasculogenesis will take place due to endothelial self-assembly around the patterned vessels, in parallel to maturation of the latter. The effect of cyclic stretch on the morphology of vessel wall lining cells in this study is in line with previous findings,^23^ where endothelial cells have been shown to elongate and orient themselves under cyclic stretch. Furthermore, the deformation of the entire vessel reflects the importance of mechanically actuated, three-dimensional *in vitro* models in research. The observed effects could also be due to an overall hydrogel deformation indicated by the deformed vessels, or the same spreading effect by cyclic stretch seen in the cells lining the walls. Taken together, we have demonstrated the critical aspect of using patterning and self-assembly techniques to create a platform for on-chip vascularization and the inclusion of cyclic stretch.

### Effect of 3D cyclic stretch on vasculogenesis

In addition to the patterned vessels, the effect of 3D cyclic stretch on endothelial vasculogenesis can be investigated. We showed that 3D cyclic stretch positively affects endothelial vasculogenesis *in vitro*. The density of the microvasculature network formed by vasculogenesis significantly increased upon cyclic stretch. Zeinali et al. reported that 3D cyclic stretch has adverse effects on sprouting angiogenesis, highlighting that formation of the sprouts from the parent vessel decreases significantly on exposure to cyclic stretch.^23^ In contrast to the latter study that focused on the effect of cyclic stretch on sprouting angiogenesis induced by VEGF, the present research investigates the effect of cyclic stretch on *de novo* vasculogenesis. It can thus be concluded that 3D cyclic mechanical stretch has differential effects on sprouting angiogenesis and on *de novo* vasculogenesis: decrease in sprouting angiogenesis and increase in vasculogenesis. To explain this, one can consider the load (stress) exerted on both constructs (patterned vessels and microvessels) using finite element simulation (Figure S13). These simulation results showed that the average stress level tolerated by the vessels depends on their size. Another explanation for the increased vasculogenesis after cyclic stretch could be due to partial endothelial-mesenchymal transition as stretch can promote the expression of smooth muscle cell markers in endothelial cells.^29^ Moreover, the inhibition of the sprouting angiogenesis and increased vasculogenesis after cyclic stretch might be related to the level of vascular maturity. The cells responsible for vasculogenesis seem to originate from EC located in the intervessel space (Figure S14). In addition, the z-projection of the raw images results in a two-dimensional representation of a three-dimensional section and may contribute to some bias. The use of more sophisticated image analysis approaches and/or software capable of 3D rendering and path tracking might rectify this.

### Impact of cyclic stretch on the transcriptomic profile of endothelial cells of patterned vessels

Our gene expression analysis showed that upon cyclic stretch expression of ANGPTL5, ANGPTL4, RND1, PLEC, IGFBP5, and PDE1A increases and expression of RHOU, Clec10A, LYVE-1, and SESN3 decreases. The ANGPTL protein family has autocrine and paracrine functions in angiogenesis, cancer progression, and metastasis. While ANGPTL5 is structurally similar to the others, its role in angiogenesis remains unclear to this date. It is mostly expressed in adipose and heart tissues. It has been associated with inflammation due to elevated levels found in obese patients with type 2 diabetes mellitus.^30^^,^^31^ The ANGPTL4 protein reduces vascular permeability and inhibits angiogenesis and inflammatory signaling in various tissues.^32^^,^^33^ Furthermore, ANGPTL4 suppresses HUVECs tube formation and proliferation.^34^ The phosphodiesterase PDE1A regulates cyclic nucleotides and calcium response.^35^ Although PDE1A activity has not been detected in endothelial cells, inhibition does affect endothelial cells and is a potential therapeutic target to limit angiogenesis in diseases.^36^ RHOU is expressed during the early stages of vasculogenesis in ECs.^37^ In addition, RHOU activation decreases the number of focal adhesions, thereby increasing migration distance.^38^ RND1 is explicitly expressed in ECs from embryonic development onward and during adulthood and promotes normal barrier function.^37^ A recent transcriptomic study with HUVECs found RND1 to be a novel Notch transcriptional target necessary for the Notch-mediated control of sprouting and migration.^39^ PLEC is a cytoskeletal crosslinking element of all types of intermediate filament subunit proteins, microtubules, and actin. PLEC-deficient endothelial cell layers were leakier and had impaired resilience to shear and strain forces.^40^ Clec10A is prominently expressed in many immune cells, and has been proposed to play an essential role in immune response initiation.^41^ Depending on the flow dynamics, ECs present a pro-inflammatory phenotype, especially under oscillatory flow.^42^ LYVE1 is a lymph vessel marker ^43^ and is also expressed in immature embryonic blood vessels.^44^ SESN3, part of the sestrin protein family, is induced by various stress conditions and regulates oxidative stress, inflammatory responses, and cell apoptosis.^45^^,^^46^ IGFBP5 is part of a family of proteins regulating the highly conserved insulin-like growth factor (IGF) signaling.^47^ Rho et al. found IGFBP5 to counteract the induction of proliferation and tube formation through VEGF in HUVECs. In their study, they concluded that IGFPB-5 expression plays a role as tumor suppressor through the inhibition of angiogenesis.^48^

The increased response to fluid shear stress as reflected by the GO:BP pathway correlates with the fluid flow induced via the deflection of the hydrogel cell culture construct. In concordance, the KEGG pathways are upregulated under stretch. The KEGG analysis also revealed decreased insulin resistance and insulin signaling pathways. A study by Shrader et al. found that insulin promoted the viability and proliferation of HUVECs.^49^ This study followed up on their previously published research on the effects of acute stretch on injured mouse skin and demonstrated proliferation of endothelial cells and upregulation of insulin and mitogenic signaling intermediates.^50^

### Conclusion and outlook

This study identifies the effects of singular or combined biomechanical and biochemical stimuli (cyclic stretch, shear forces, and ECM hydrogel stiffness) on vascularization in a microfluidic vascular system. It shows that even minor variations of those stimuli (e.g., slight difference of the ECM stiffness) can affect the formation of complex microvasculature. The development of an *in vivo*-like model that mimics such a complex microvasculature, including large and small vessels, is an important tool for OOC and tissue engineering research. The presented device could also be adapted for several tissue barrier models through epithelial cell seeding on top or inside the second templated channel. In addition, embedding of organoids between the vessels post-gelation using a technique proposed by Brassard et al.^51^ could give new insights into organoid vascularization studies.^52^ Furthermore, tumor extravasation^53^^,^^54^ is another research field that could utilize this *in vitro* device with two main parallel vessels. With the inclusion of mesenchymal cells into the hydrogel solution itself,^55^ perfusable anastomotic vessels could be created, therefore artificially replicating hierarchical vessel structures. After initial validation of the device and its open-access design, a polystyrene-based plate variant could allow for high-throughput production and screening.^56^ With the incorporation of pluripotent stem cells, this device could not only enable personalized medicine but also reach toward regenerative medicine.^57^

### Limitations of the study

This study employs a unique organs-on-chip device to investigate the combined or singular effects of different biomechanical or biochemical stimuli and represents a starting point for research on vascular development. As such, it employed only one cell type (HUVECs), whereas blood vessels *in vivo* are made of several different cell types. Furthermore, the three-dimensional hydrogel compartments with the cells were imaged using fluorescent laser scan microscopy, but the analysis of sprouting angiogenesis and vasculogenesis was conducted on projected two-dimensional images. Future studies with the presented chip should therefore account for these limitations, namely including different cell types and three-dimensional image analysis.

## STAR★Methods

### Key resources table

**Table:** 

REAGENT or RESOURCE	SOURCE	IDENTIFIER
**Antibodies**		

CD31 (PECAM-1) (89C2) Mouse mAb	Cell Signaling Technologies	Cat#3528; RRID: AB_2160882
Invitrogen Anti-Mouse IgG (H + L) Secondary Antibody, Superclonal™	ThermoFisher Scientific	Cat#A28175; RRID: AB_2536161
F-actin/Phalloidin 670	Invitrogen	Cat#PHDN1-A
Hoechst 33342	Invitrogen	Cat#H3570

**Chemicals, peptides, and recombinant proteins**		

NucleoSpin RNA XS kit	Machery-Nagel	Cat#740902.50
Triton X-100	Sigma-Aldrich	Cat#X100
VEGF165	Miltenyi	Cat#130-109-384
Fibrinogen	Sigma-Aldrich	Cat#F8630-1g
Thrombin	Sigma-Aldrich	Cat#T4648-10KU
TrypLE express	Gibco	Cat#12604013

**Experimental models: Cell lines**		

HUVEC	Gibco	Cat# C01510C;

**Software and algorithms**		

ImageJ	National Institutes of Health	www.ImageJ.net
Graphpad Prism 9	GraphPad Software Inc.	www.graphpad.com
Python 3	Python Software Foundation	www.python.org

### Resource availability

#### Lead contact

Further information and requests for resources and reagents should be directed to and will be fulfilled by the lead contact, Oliver T. Guenat (olivier.guenat@unibe.ch).

#### Materials availability

This study did not generate new unique reagents.

### Experimental model and subject details

Human umbilical vein endothelial cells (HUVEC, Gibco), between passages 4 to 6, were cultured in standard cell culture flasks with endothelial growth medium-2 (EGM2, Lonza) until 90% confluency was reached. TrypLE express was then applied for 3-5 min at 37°C to collect the cells from the flask and subsequently spun down. Cells were resuspended in 1 mL of medium and counted using an automated cell counter (LUNA-II, Logos Biosystems). For seeding in the chips, a concentration of 2x10^7^ cells/ml was used, either for direct seeding of the channels or later dilution with fibrinogen solution (final concentration of 1x10^7^ cells/ml) to fabricate the cell-laden fibrin hydrogel. No ethical approval was necessary for this study, as these cells are commercially available and are pooled cells (multiple donors, no sex).

### Method details

#### Chip production

The device consists of three layers with a membrane between the bottom and middle layers. Bottom and middle layer molds were designed using SOLIDWORKS software (Dassault Systèmes) and produced through stereolithography by PROFORM (Freiburg, Switzerland). Polydimethylsiloxane (PDMS, Sylgard 184, Dow Corning) was mixed according to the manufacturer’s instructions at a 10:1 base to curing agent ratio and poured into the molds. Molds with the liquid silicone were later degassed, and a polyester foil was carefully placed on top to reduce air bubbles. After clamping the molds between two polystyrene plates, the PDMS was cured at 60°C overnight. Subsequently, the PDMS layers were removed and post-processed, including trimming borders, punching medium wells and pneumatic accesses with dermatology biopsy punchers, and cleaning the PDMS layers with standard office supply tape. Two 100 μm diameter rods (Tsubame (J00378A)) were placed in the channels and rod removal guides in the post-processed middle layer. The PDMS membrane between the bottom and the intermediate layer was spin-coated on a silicon wafer covered by a polyester foil at 600 RPM for 60 s and cured at 60°C overnight. The resulting 100 μm thick PDMS membrane was then oxygen plasma bonded (Harrick Plasma, USA) onto the middle layer facing the side with the rods. The bottom chip layer was oxygen plasma bonded on a generic microscope glass slide. The polyester foil covering the membrane was removed. The middle layer with the membrane was immediately put on top of the bottom layer, ensuring that no air bubbles or dust particles were trapped between the layers. Afterward, using 8- and 6-mm dermatology biopsy punchers, small PDMS rings were created and oxygen plasma bonded over the central well chamber of the middle layer.

For cyclic stretch (CS) experiments, the chips were put into Petri dishes with a drilled hole as an access port for tubing and connected to an external electro-pneumatic setup (house-made setup and Flow EZ™ (Fluigent)), with settings described in,^23^ where CS^low^ settings correspond to a linear strain of 8–12%. For static experiments, chips were put into unaltered Petri dishes. Additional small containers with deionized water reservoirs for humidity were added to the Petri dishes. For all cell experiments, chip and container sterilization was conducted using an ozone chamber (CoolCLAVE, Genlantis) and UV sterilization (UV-C, 30 min) under a laminar flow hood.

#### Hydrogel fabrication and chip seeding

Fibrinogen (bovine plasma, Sigma) at concentrations of 20 and 30 mg/mL was mixed with endothelial basal medium-2 (EBM2, Lonza) containing thrombin (2U, bovine plasma, Sigma) to produce 10 and 15 mg/mL fibrin hydrogels. In the vasculogenesis model, cells were suspended in the EBM2-thrombin solution, and 15 mg/mL fibrin hydrogel solutions were used. After filling the chips with mixed precursor solution, gelation proceeded for 5 min at room temperature, and then for 25 min at 37°C. Next, the rods were removed with a swift, unidirectional motion and the seeding of the channels with cells (2x10^7^ cells/ml) was conducted. 9 μL of cell suspension per channel was required for filling, ensuring that the solution didn’t spread into the opposing medium wells (Figure S2). The chips were kept in culture for eight days for the sprouting angiogenesis and five days for the vasculogenesis model. Treatments with VEGF^165^ (Vascular Endothelial Growth Factor, Miltenyi Biotec) were done at a 50 ng/mL concentration in culture medium (EGM2). The culture medium was changed daily, while occasional clogging of the channels from cell clusters during the first days in culture was removed by pushing 10 μL of medium through the channels. The medium exchange followed the same procedure, where the first two neighboring wells were filled, and after 5-15 min, the medium equilibrated between the two pairs of opposing medium wells. Finally, the initially filled medium wells were once again filled.

#### Immunostaining and imaging

Chips were washed 3 times with PBS, fixed for 15 min in 4% paraformaldehyde (Sigma), and washed 3 times again with PBS. Afterward, cells were permeabilized for immunostaining with 0.01% of Triton X-100 (Sigma) for 10 min, followed by another washing cycle and the addition of 2% BSA in PBS as a blocking buffer. Mouse anti-PECAM-1 (mouse, Cell Signaling) at a 1:200 dilution in PBS was added for 24 h and incubated at 4°C. Then, chips were washed once more, and the second antibody (anti-mouse Alexa Fluor 488, Molecular Probes) as well as the dyes (F-actin and Hoechst, Invitrogen) were added and incubated for 12 h at 4°C and washed. The middle chip layer (with the membrane) was then detached and placed on a coverslip with a drilled hole to increase the working distance at the microscope. Imaging was performed on a Zeiss LSM 710. Whole vessel scans were done at a magnification of 10× with tilescan and z stack mode enabled. Close-ups of regions of interest, the intervessel space, and the vessel walls were conducted using a magnification of 20× in tilescan and z stack mode.

#### Gene expression: Next-generation sequencing

12 chips were used for this analysis, 6 per condition (static and stretch). Since HUVEC cells from the same batch and from the same passage were used, the biological replicate is 1, however the technical replicate equals to 6. Only cells from the main patterned vessels were collected for the gene expression analysis from within the vasculogenesis model. Using the lysis kit NucleoSpin RNA XS (Macherey-Nagel), the solution was pushed through each vessel per chip three times, until all cells from the main vessel wall were collected. The RNA obtained from two chips were pooled, as the amount of RNA from a single chip was not sufficient for the NGS study, resulting in three samples per condition for the final analysis. The same kit was used for RNA purification. Next-generation sequencing was conducted by Microsynth (Switzerland). Library preparation employed Illumina TruSeq, stranded, poly-A enriched RNA libraries, and sequencing on Illumina NextSeq, v2.5, 1x75 bp, demultiplexin, and trimming of Illumina adaptor residuals. For standard differential gene expression analysis, human reference genome hg38 was used. The nf-core/rnaseq (v3.5)^58^ workflow was used for generating expression matrix. In this workflow, Trim Galore (v0.6.7) was used to trim the 75-bp single-end reads prior to genome alignment. Read alignment and gene counts were performed using STAR(v2.7.6a)^59^ and RSEM(v1.3.1)^60^ with Hg38 primary reference genome. The gene count matrix was imported into R(v4.1.2)^61^ and low expressed genes were filtered out. 13,863 genes were retained for differentiated expressed gene analysis. Wald test in DEseq2(v1.34.0)^62^ was performed to identify deregulated genes between static and stretch. Shrunken log2 fold changes (LFC) using apeglm^63^ was performed to generate more accurate log2 fold change estimates. 92 up-regulated and 62 down-regulated genes were identified with padj <0.05 and LFC >0.5. Further gene Set Enrichment Analysis of GO Biological Process ontology and KEGG were done using gseGO and gseKEGG in clusterProfiler(v4.2.2)^64^^,^^65^ R package.

### Quantification and statistical analysis

Raw image files were analyzed in Fiji/ImageJ^66^ depending on the experimental model (angiogenesis or vasculogenesis sections). For vessel dimensions and sprouts measurements, 10x images were z-projected in maximum intensity mode, and the length/amount of the sprouts were measured manually. Cell parameters (area, length, and orientation) were measured manually on 20x scans of the main vessel walls, using three different locations of one vessel per chip. In contrast, nuclei parameters were measured with the Particle Analyzer from Fiji in an automated approach, using three distinct parts of the main vessel wall (left, middle, right) from 10x scans. The intervessel network was analyzed using 20x images tilescans, of which a central square of the same size for all images was cropped out. Segmentations were performed using the ImageJ WEKA plugin^67^ and subsequent skeletonizations were analyzed with the Analyze Skeleton plugin.^68^ For circularity measurements on the patterned vessel, a reslice of three parts of the 10x z-stacks in y-direction was performed, and subsequently 2D projected in maximum intensity mode. This resulted in representative cross-section images of both vessels per chip over the whole vessel length. Graphs and statistical analysis were conducted using GraphPadPrism 9.2 Software. For comparison of three or four conditions, ordinary one-way ANOVA with Tukey’s multiple comparisons tests were applied. For comparisons of two conditions, unpaired t-tests were applied. Confidence indicators represent the following p values: p value <0.0001: ∗∗∗∗; p value <0.001: ∗∗∗; p value <0.01: ∗∗; p value <0.05: ∗ p value >0.05: ns. N represents number of independent experiments, n represents number of chips, and can be found in the figure legends.

## Data Availability

• All data reported in this paper will be shared by the lead contact upon request. All original code is available in this paper’s supplemental information. Any additional information required to reanalyze the data reported in this paper is available from the lead contact upon request. • Gene analysis data can be accessed under https://doi.org/10.48620/181
