# Supplementary material for: Effects of biomechanical and biochemical stimuli on angio- and vasculogenesis in a complex microvasculature-on-chip
Source: iScience. 2023 Feb 13;26(3):106198. doi: 10.1016/j.isci.2023.106198 (PMC9985038; doi:10.1016/j.isci.2023.106198)
Supplement: Document S1. Supplementary methods, Figures S1–S14, and Table S1 [file mmc1.pdf]

iScience, Volume *26*

Supplemental information

Effects of biomechanical and biochemical stimuli on angio- and vasculogenesis in a complex microvasculature-on-chip

Dario Ferrari, Arunima Sengupta, Lyong Heo, Laszlo Pethö, Johann Michler, Thomas Geiser, Vinicio A. de Jesus Perez, Wolfgang M. Kuebler, Soheila Zeinali, and Olivier T. Guenat

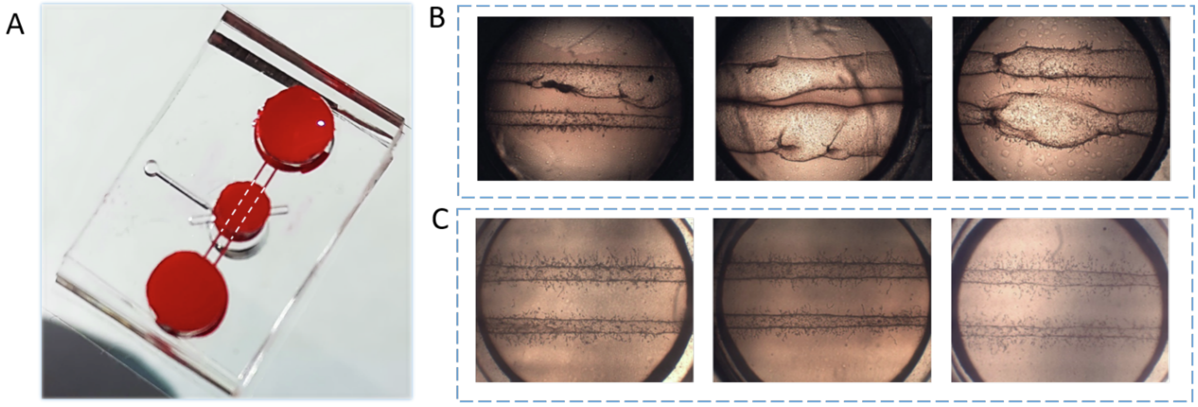

Figure S1, related to Figure 1: Design improvements of the microfluidic chip

A) Picture of the first microfluidic chip. B) Representative outcomes of the parallel vessels obtained with the first design. C) Representative outcomes of the parallel vessels obtained with the second design.

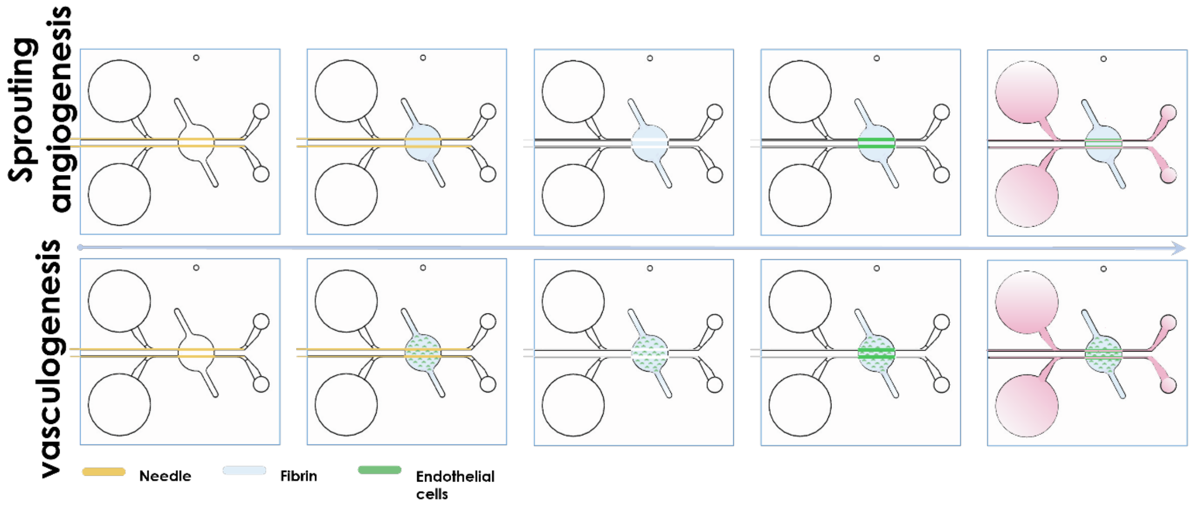

Figure S2, related to Figure1: Process steps to cell seeding on chip

Process based on the two different approaches, angiogenesis (top) and vasculogenesis (bottom). From left to right: the finished chip pre-cell culture with the two needles (yellow), the filling of the central well chamber with fibrin hydrogel, the removal of the needles leaving empty channels inside the hydrogel, the patterning of these empty channels through seeding with endothelial cells, the filling of the medium wells with cell culture medium.

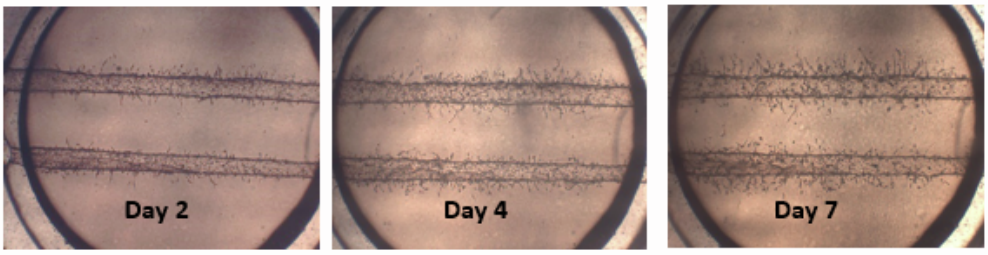

Figure S3, related to Figure 1: Real-time observation of the vessel endothelialisation and sprouting

Pictures of the remodeling and sprouts growth of the parallel vessels in function of time. Cell seeding took place on Day 1.

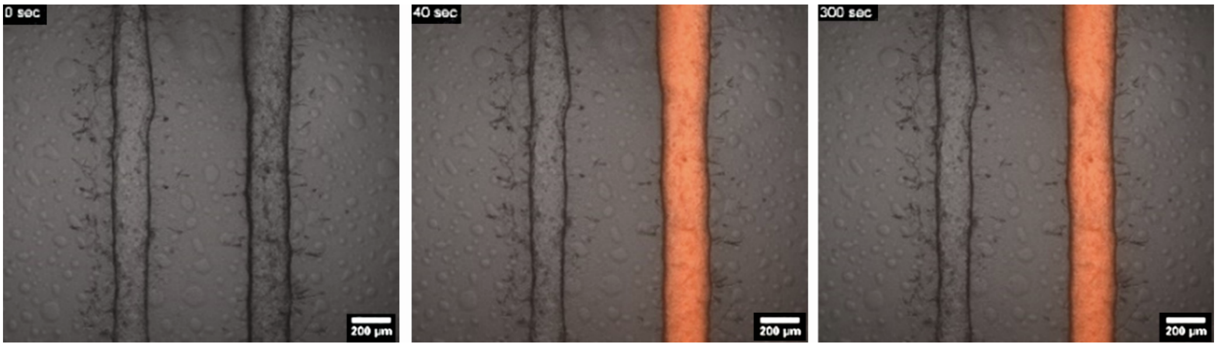

Figure S4, related to Figure 1: Independently perfusable channels

Exemplified through the use of fluorescent RITC (Rhodamin B Iso-Thiocyanate), 1 mg/ml in PBS, the patterned vessels can be independently perfused.

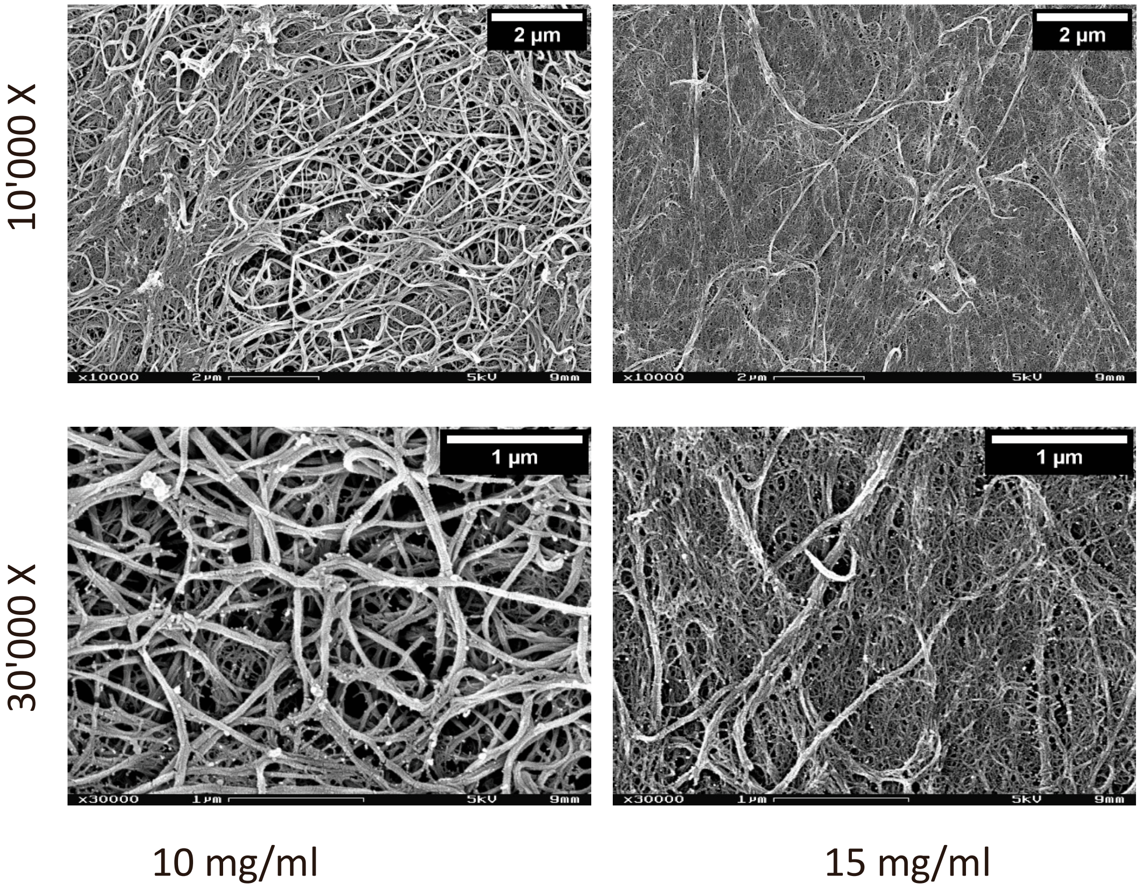

Figure S5, related to Figure 3: Scanning Electron Microscope (SEM) images of the hydrogels

The two different fibrin hydrogel concentrations used in this study were imaged at different magnifications. Top row: 10’000 x magnification, bottom row 30’000 x magnification. Left column: 10 mg/ml fibrin hydrogel, right column: 15 mg/ml fibrin hydrogel.

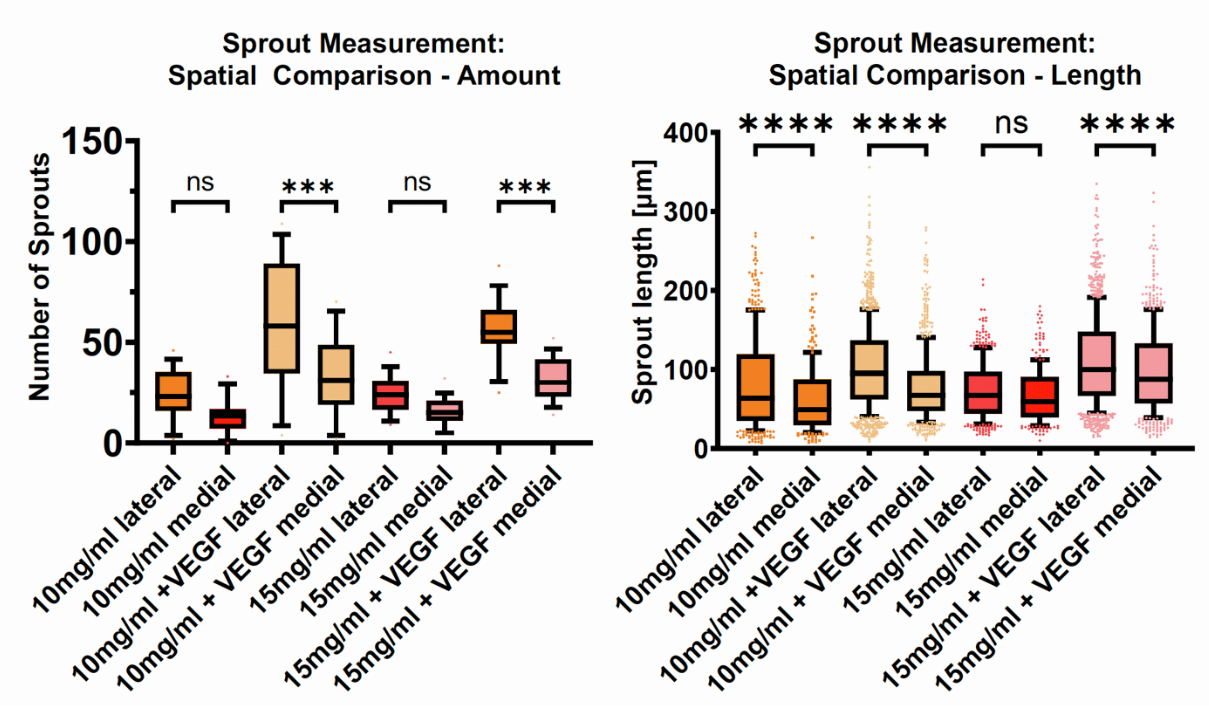

Figure S6, related to Figure 3: Spatial positioning of sprouts

Measurements of sprouts separated by their location relative to the main patterned vessel (lateral vs medial) under different conditions. The medial position is the position between the two vessels, the lateral is next to the channels. (ordinary one-way ANOVA, p-value > 0.05: ns, p-value < 0.0001: ****; p-value < 0.001: ***; p-value < 0.01: **; p-value < 0.05: *, N=3, total n=9/condition).

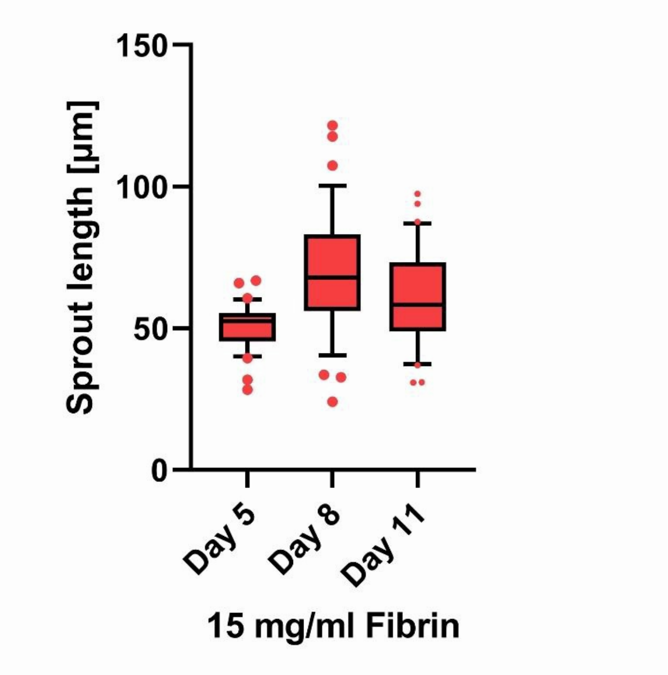

Figure S7, related to Figure 3: Sprout measurement over time

The length of sprouts from chips fixed at different times over the culture time (N=1, n=3, whiskers:10-90 percentile).

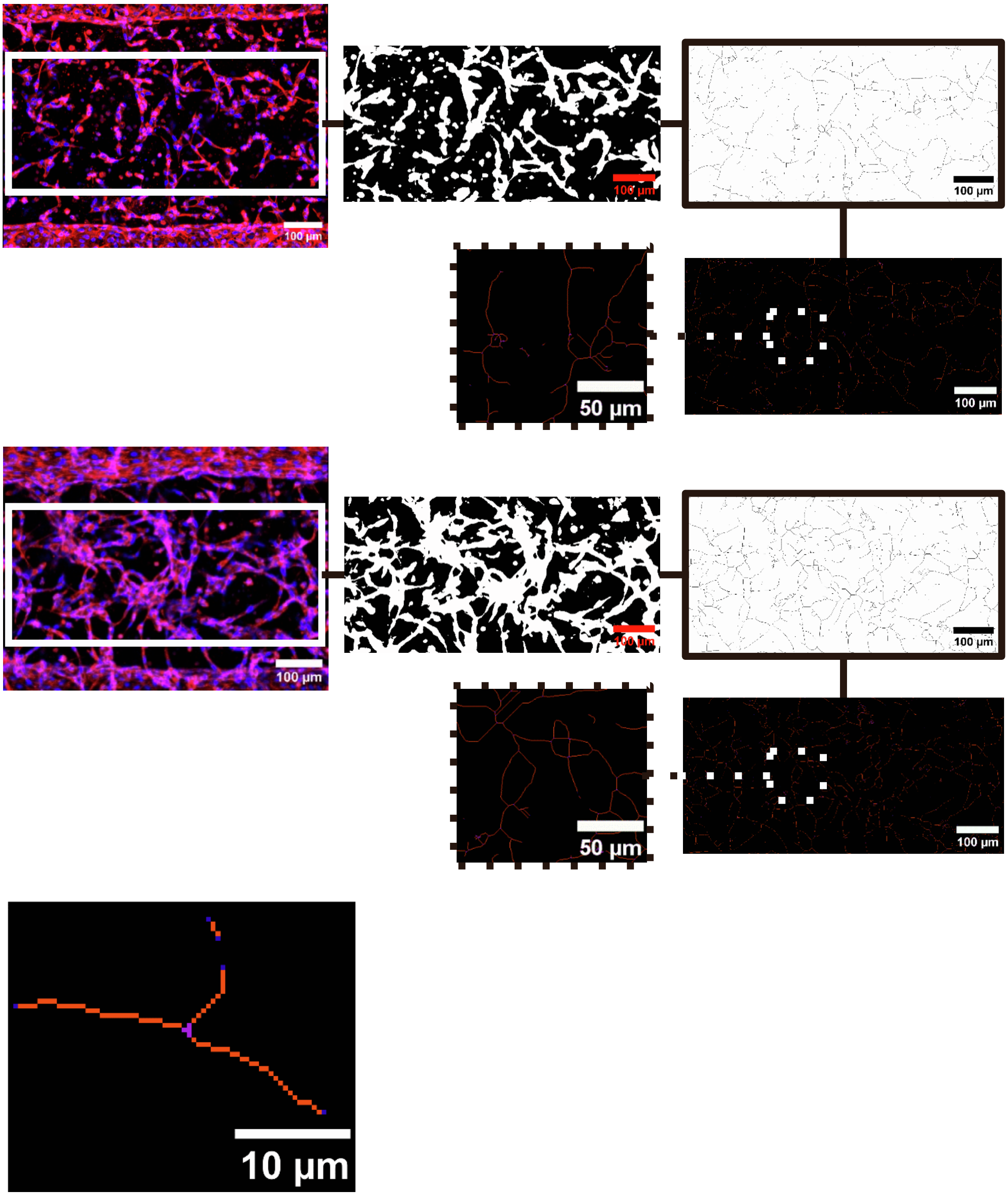

Figure S8, related to Figure 4: Image processing of the intervessel space

Top cluster and middle cluster show representative images under static and stretch condition, respectively. First, a central rectangle was cut from the intervessel space, followed by segmentation with WEKA plugin. Subsequently, skeletonization was performed, then analyzed. The images with black backgrounds show the results of the analysiation of the skeletonized image. The bottom image shows a close-up example, with end pixels (blue), junction pixels (violet), and branch pixels (orange).

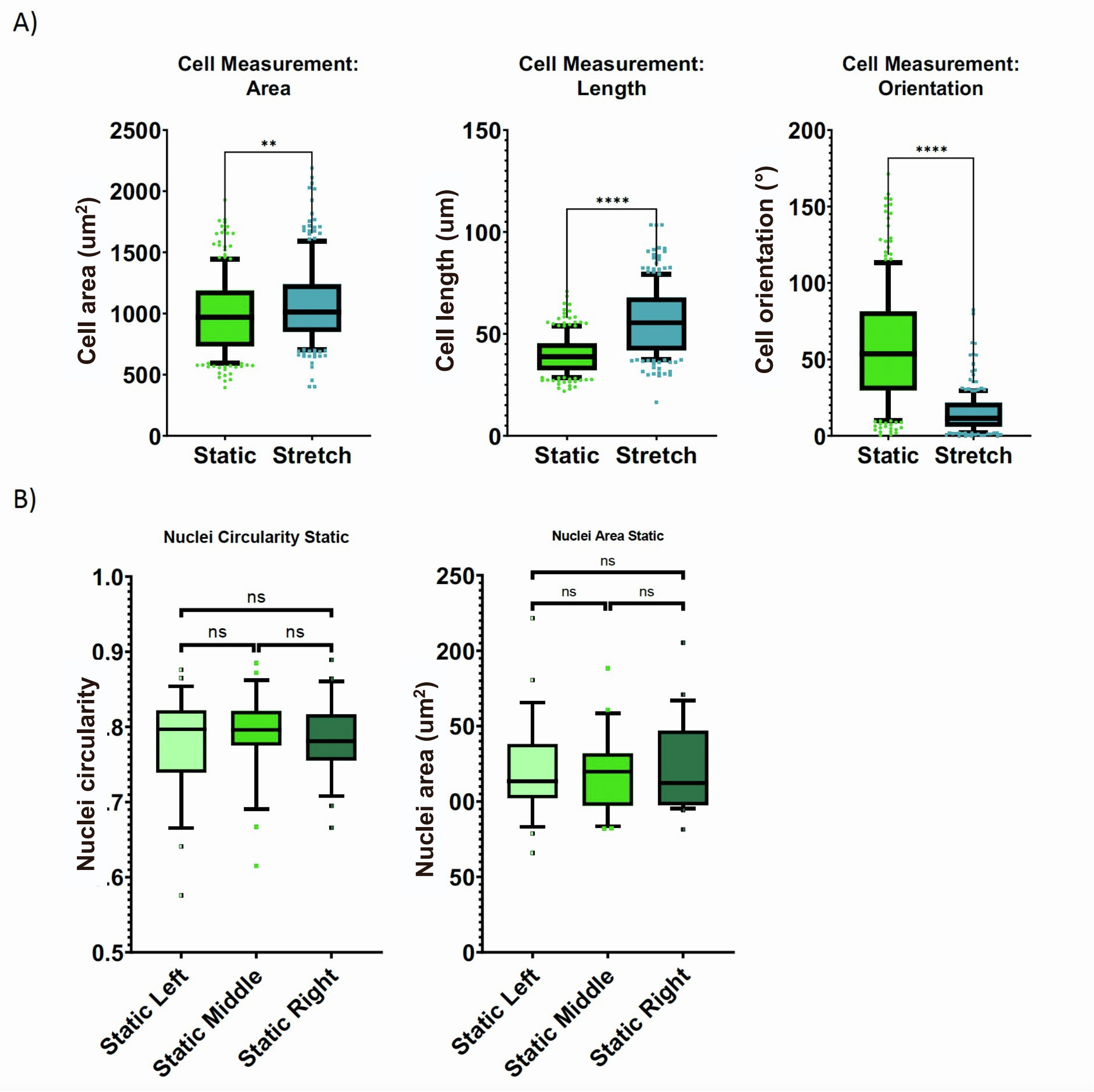

Figure S9, related to Figure 5: Parameters of the cells lining the vessel walls

A) Manual measurements on cell lining the vessel wall under different conditions. The manual measurements on the whole cell were initially conducted, replaced with later automated measurements on the nuclei (unpaired t-test, p-value < 0.0001: ****; p-value < 0.001: ***; p-value < 0.01: **; p-value < 0.05: *, N=2, n=4). B) Positional comparison of nuclei morphology under static condition (whiskers: 10-90 percentile, ordinary one-way ANOVA, p-value < 0.0001: ****; p-value < 0.001: ***; p-value < 0.01: **; p-value < 0.05: *, N=3, total n=12).

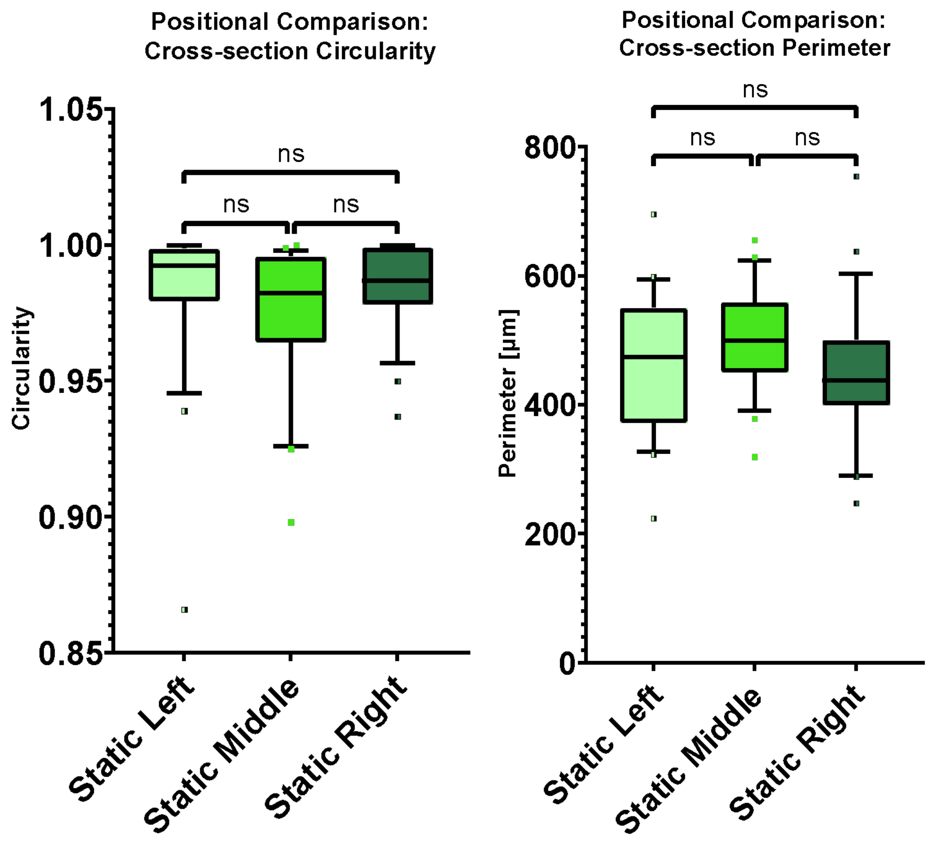

Figure S10, related to Figure 6: Vessel cross-section parameters at different locations

Depending on the localisation of the cross-section measurements as shown in Fig. 5C, results were categorized in 'left', 'middle', and 'right' sections. To the left, the circularity measurements for static condition are shown. To the right, the perimeter measurements for static condition are shown. (Unpaired t-test, p-value < 0.0001: ****; p-value < 0.001: ***; p-value < 0.01: **; p-value < 0.05: *, N=3, n=14).

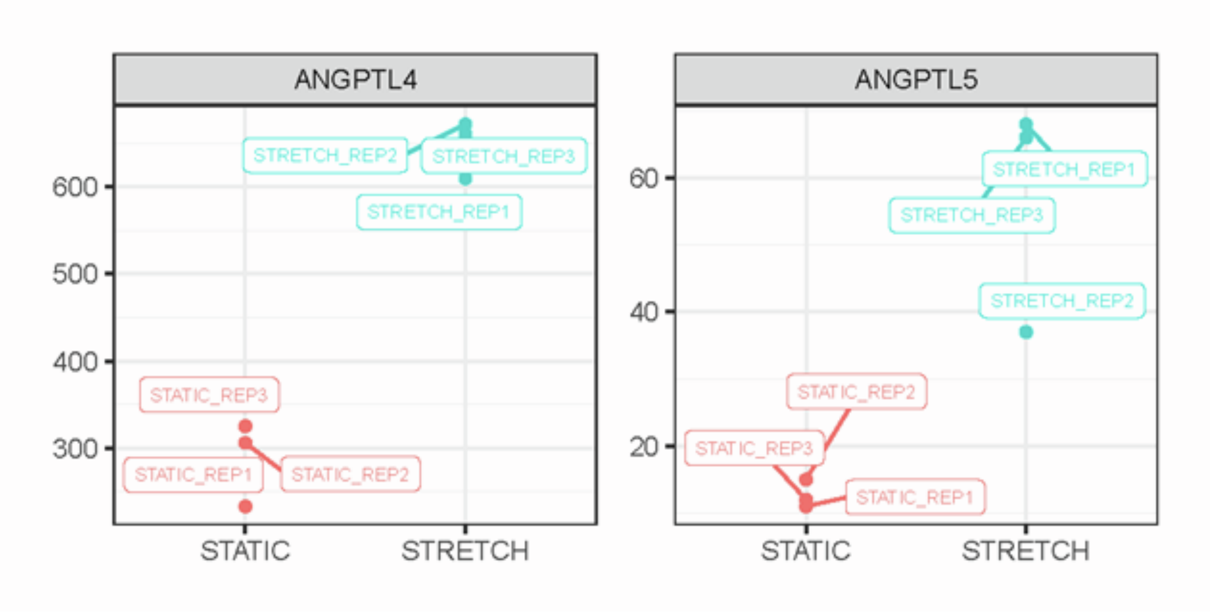

Figure S11, related to Figure 7: Gene expression analysis of ANGPTL4+5.

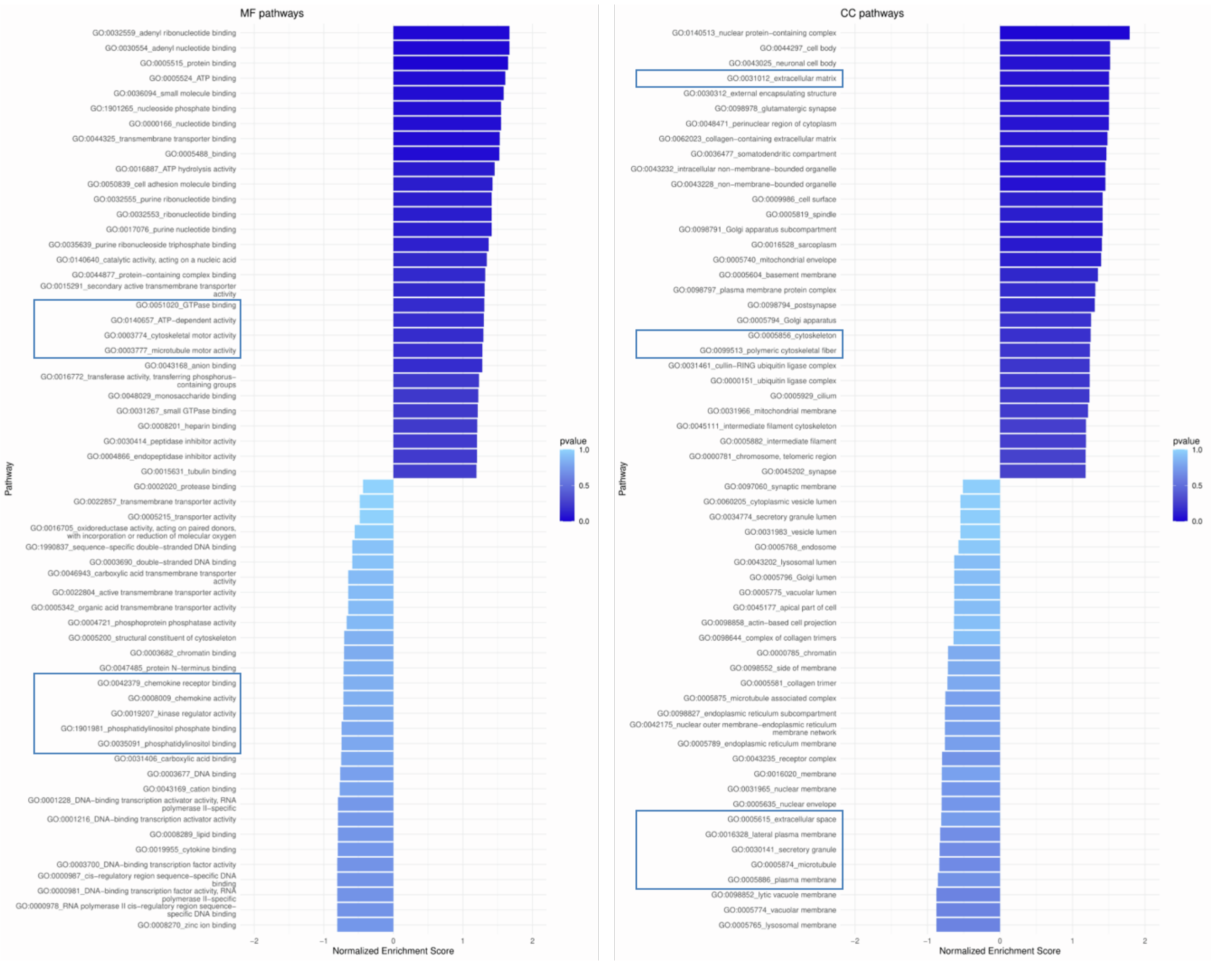

Figure S12, related to Figure 8: Biological pathway analysis

GO:MF pathways (left), and GO:CC pathways (right). Pathways of especial interest are highlighted with frames.

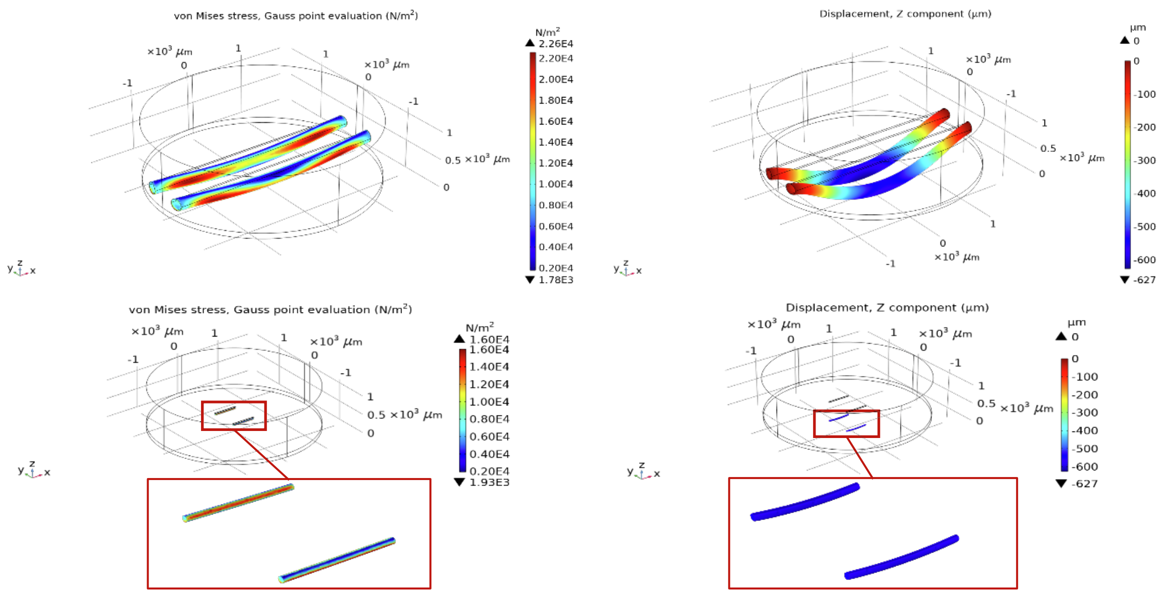

Figure S13, related to Discussion: Vessel strain simulation

The impact of 3D mechanical stretch on small and large vasculatures. Displacement field in Z direction and distribution of the von Mises stresses on vasculatures walls are shown here. Under the same magnitude of stretch applied on the membrane/hydrogel layer, the maxium von Mises stress tolerated by large vasculature is at least two times higher than the small vasculature.

Comsol Multiphysics Simulation: Effect of 3D mechanical stretch on small and large vasculatures

COMSOL Multiphysics®6 was used to perform finite element simulation of the 3D vasculatures under mechanical stretch. 3D structure of the multilayer composed of PDMS membrane and hydrogel layer incorporating one vasculature (large or small) was drawn using geometry toolbar of the software. Multilayer was adopted as linear elastic material within solid mechanics interface. This simulation is carried out to better understand the effect of the mechanical stretch applied on the hydrogel layer on small and large vasculatures. Small vasculature refers to vessels which are created by sprouting angiogenesis or vasculogenesis. Large vasculature refers to vessels which are created by patterning. In one simulation, the hydrogel layer incorporated a large vasculature with diameter of 200 μm and length of 3.5 mm. In the other simulation, the hydrogel incorporates a candidate small vasculature with diameter of 30 μm and length of 500 μm. To make sure that the results are comparable, both vasculatures are centered in the same location and the same magnitude of mechanical stretch is applied on the flexible membrane at the bottom of the hydrogel layer. To investigate the impact of the mechanical stretch, two parameters are considered: displacement field of the vasculatures in the Z direction (the direction in which stretch is applied) and distribution of the von Mises stress on the vasculatures walls. In regards to the boundary conditions that each of the vasculatures have, the nature of the displacement fields are different. In the simulation similar to experimental platform, the large vessel is anchord to the cyclindrical chamber from its ends and has non-homogeneous displacement. However, small vasculature is not restricted and has uniform displacement upon stretch. As both vasculatures are exposed to 3D mechanical stretch, the distribution of the stress is non-homogeneous within the vasculatures walls. Maximum von Mises stress is a good measurement parameter to compare level of the stress tolerated by each vasculature upon 3D stretch. As our simulation results show, under the same magnitude of stretch applied on the membrane/hydrogel layer, the maximum von Mises stress tolerated by large vasculature is at least two times higher than the small vasculature.

Static:

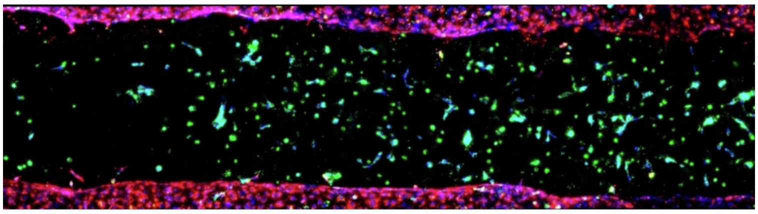

Static: Whole scan of the intervessel space

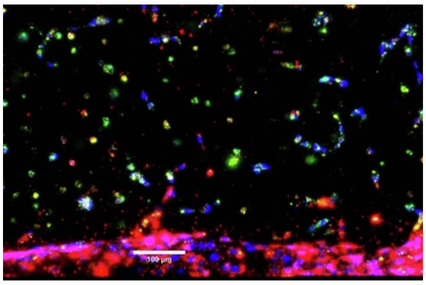

Static: Close-up on sprout

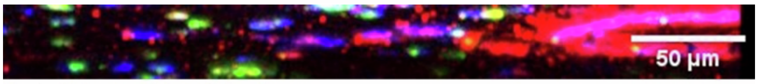

Static: Orthogonal view of the close-up

Stretch:

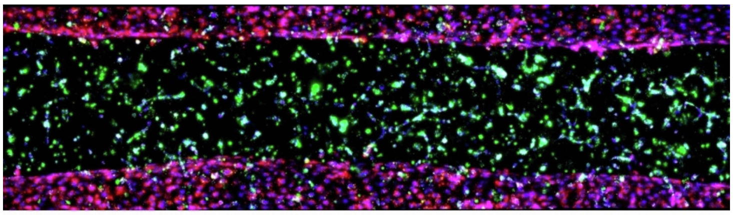

Stretch: Whole scan of the intervessel space

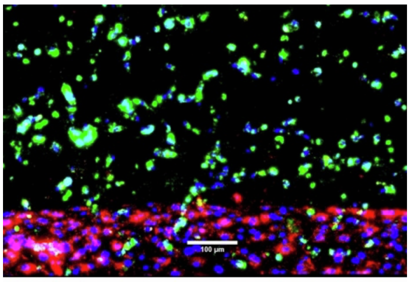

Stretch: Close-up on sprout

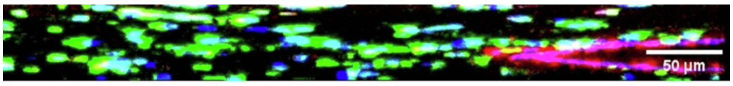

Stretch: Orthogonal view of the close-up

Figure S14, related to Discussion: Origin of the vessel network

An experiment using PKH-membrane-stains was performed to identify whether the formed network, derives from sprouting growing from the patterned vessels or from the self assembling of endotelial cells in the gel. One can observe that the cells suspended in the hydrogel (green) dominate the intervessel space and the cells seeded inside the preformed channels (red) don't reach into this space. With the orthogonal view focusing on a sample protrusion of the main vessel, one can depict a clear difference between the static and stretch condition, where the latter shows no red signal outside the main vessel wall. This, combined with previous work finding angiogenic sprouting breaking off under strained condition[1], lets us deduce, that the origin of the formed network is mostly from the suspended cells.

---

[1] Zeinali, S., et al. (2021). Remodeling of an in vitro microvessel exposed to cyclic mechanical stretch. APL Bioeng. 5, 026102. 10.1063/5.0010159.

Supplementary Table 1 , related to Figure 7: Top up- and down-regulated differentially expressed genes in stretched endothelial cells compared to the static cells on-chip. The expression values are log_2fold changes (>1) between static and stretch samples.

Gene	Full gene name	Up/downregulated	Fold change
IGFBP5	Insulin like growth factor binding protein 5	Up	2.2
PDE1A	Phosphodiesterase 1A	Up	2.07
ANGPTL5	Angiopoietin Like 5	Up	1.87
ANOS1	Anosmin 1	Up	1.76
RND1	Rho family GTPase 1	Up	1.56
TSPAN2	Tetraspanin 2	Up	1.39
FOSB	FosB proto-oncogene, aka AP-1	Up	1.36
ANGPTL4	Angiopoietin Like 4	Up	1.19
GJA5	Gap junction protein alpha 5, aka connexin 40	Up	1.04
CLEC10A	C-Type Lectin Domain Containing 10A	Down	1.72
LINC00622	Long intergenic non-protein coding RNA 622	Down	1.68
HTR2B	5-Hydroxytryptamine Receptor 2B	Down	1.51
LYVE1	Lymphatic vessel endothelial hyaluronan receptor 1	Down	1.5
GDF3	Growth Differentiation Factor 3	Down	1.26
ENPP2	Ectonucleotide Pyrophosphatase/Phosphodiesterase 2	Down	1.24
ACP5	Acid Phosphatase 5	Down	1.14
NUPR1	Nuclear Protein 1	Down	1.14
SPTA1	Spectrin Alpha Erythrocytic 1	Down	1.12
BMPER	BMP Binding Endothelial Regulator	Down	1.05
SESN3	Sestrin 3	Down	1.05
N4BP2L1	NEDD4 Binding Protein 2 Like 1	Down	1.04
TTC39A	Tetratricopeptide Repeat Domain 39A	Down	1.03
LOC100128288	Uncharacterized	Down	1.02
RHOU	Ras Homolog Family Member U	Down	1.01

Supplementary Methods: Basic ImageJ macros used in this paper, related to STAR Methods

```
//-----------------------------------------------------------------------------------------------------------
//General batch processing of one folder with images (respective functions see later)
//-----------------------------------------------------------------------------------------------------------
input = getDirectory("Choose an input directory");
output = getDirectory("Choose an output directory");

processFolder(input);

function processFolder(dir) {
	list = getFileList(dir);
	for (i=0; i<list.length; i++) {
		if(endsWith(list[i], ".czi")) {
//add the file ending for your images
			processFile(dir, output, list[i]);
		} else if(endsWith(list[i], "/") && !matches(output, ".*" + substring(list[i], 0,
lengthOf(list[i])-1) + ".*")) {
		processFolder(""+dir+list[i]);
		} else {
		print(dir + list[i]);
		}
	}
}

//insert respective function (below) to batch process here

close("*");

//-----------------------------------------------------------------------------------------------------------
//Function for Enhancing Contrast, Create Composite, z-Project
//-----------------------------------------------------------------------------------------------------------
function processFile(inputFolder, output, file) {
	open(inputFolder + file);
	title = getTitle();
	run("Split Channels");
	three = "C3-" +title;
	two = "C2-" +title;
	one = "C1-" + title;

	selectWindow(three);
	run("Enhance Contrast...", "saturated=0.3 normalize process_all");
	run("Despeckle", "stack");
	selectWindow(two);
	run("Enhance Contrast...", "saturated=0.3 normalize process_all");
	run("Despeckle", "stack");
	selectWindow(one);
	run("Enhance Contrast...", "saturated=0.3 normalize process_all");
	run("Despeckle", "stack");

	run("Merge Channels...", "c1=["+three+"] c2=["+two+"] c3=["+one+"] create");
	run("Z Project...");
```

```
        saveAs("tiff", output + file);
        saveAs("Jpeg", output + file);    //for preview on Windows
        close(file);
        close("*");
}

//----------------------------------------------------------------------------------------------------------
//Function for reslicing (vessel cross-section), 3 distinct regions
//----------------------------------------------------------------------------------------------------------
function processFile(inputFolder, output, file) {
        open(inputFolder + file);
        title = getTitle();
        run("Split Channels");
        three = "C3-" +title;
        two = "C2-" +title;
        one = "C1-" + title;

        selectWindow(three);
        run("Enhance Contrast...", "saturated=0.3 normalize process_all");
        run("Despeckle", "stack");
        selectWindow(two);
        run("Enhance Contrast...", "saturated=0.3 normalize process_all");
        run("Despeckle", "stack");
        selectWindow(one);
        run("Enhance Contrast...", "saturated=0.3 normalize process_all");
        run("Despeckle", "stack");
        run("Merge Channels...", "c1=["+three+"] c2=["+two+"] c3=["+one+"] create");
        rename(title);

// 1st region, vessel left

        res1 = "Reslice of "+ title;
        makeRectangle(100, 0, 41, 1990);
        run("Reslice [/]...", "output=5.000 start=Left");
        max1 = "MAX_" + res1;
        run("Z Project...", "projection=[Max Intensity]");

        selectWindow(res1);
        saveAs("Tiff", output + "1." + res1);
        close();
        selectWindow(max1);
        saveAs("Tiff", output2 + "1." + max1);
        close();

// 2nd region, vessel middle

        res2 = "Reslice of " + title;
        makeRectangle(1477, 0, 41, 1990);
        run("Reslice [/]...", "output=5.000 start=Left");
        max2 = "MAX_" + res2;
        run("Z Project...", "projection=[Max Intensity]");

        selectWindow(res2);
```

```
        saveAs("Tiff", output + "2." + res2);
        close();
        selectWindow(max2);
        saveAs("Tiff", output2 + "2." + max2);
        close();

// 3rd region, vessel right

        res3 = "Reslice of " + title;
        makeRectangle(2860, 0, 41, 1990);
        run("Reslice [/]...", "output=5.000 start=Left");
        max3 = "MAX_" + res3;
        run("Z Project...", "projection=[Max Intensity]");

        selectWindow(res3);
        saveAs("Tiff", output + "3." + res3);
        close();
        selectWindow(max3);
        saveAs("Tiff", output2 + "3." + max3);
        close();

        close(file);
        close("*");
}

//----------------------------------------------------------------------------------------------------------------
//Function for substack creation of vessel wall, for nuclei analysis of 3 locations per vessel
//----------------------------------------------------------------------------------------------------------------
function processFile(inputFolder, output, file) {
        open(inputFolder + file);
        title = getTitle();
        print(title);
        run("Split Channels");
        three = "C3-" +title;
        two = "C2-" +title;
        one = "C1-" + title;

        selectWindow(three);
        close();
        selectWindow(two);
        close();
        selectWindow(one);
        run("Enhance Contrast...", "saturated=0.3 normalize process_all");
        run("Despeckle", "stack");

        makeRectangle(50, 500, 200, 50);
        waitForUser();
        run("Make Substack...");
        run("Z Project...", "projection=[Max Intensity]");
        saveAs("tiff", output + "V1P1_" + title);
        close();

        selectWindow(one);
```

```
        makeRectangle(1300, 500, 200, 50);
        waitForUser();
        run("Make Substack...");
        run("Z Project...", "projection=[Max Intensity]");
        saveAs("tiff", output + "V1P2_" + title);
        close();

        selectWindow(one);
        makeRectangle(2500, 500, 200, 50);
        waitForUser();
        run("Make Substack...");
        run("Z Project...", "projection=[Max Intensity]");
        saveAs("tiff", output + "V1P3_" + title);
        close();

        selectWindow(one);
        makeRectangle(50, 1300, 200, 50);
        waitForUser();
        run("Make Substack...");
        run("Z Project...", "projection=[Max Intensity]");
        saveAs("tiff", output + "V2P1_" + title);
        close();

        selectWindow(one);
        makeRectangle(1300, 1300, 200, 50);
        waitForUser();
        run("Make Substack...");
        run("Z Project...", "projection=[Max Intensity]");
        saveAs("tiff", output + "V2P2_" + title);
        close();

        selectWindow(one);
        makeRectangle(2500, 1300, 200, 50);
        waitForUser();
        run("Make Substack...");
        run("Z Project...", "projection=[Max Intensity]");
        saveAs("tiff", output + "V2P3_" + title);
        close();
        selectWindow(one);
        close(file);
        close("*");
}

//--------------------------------------------------------------------------------------------------------------------
//Function for nuclei analysis
//--------------------------------------------------------------------------------------------------------------------
function processFile(inputFolder, output, file) {
        open(inputFolder + file);
        title = getTitle();
        print(title);

        run("Unsharp Mask...", "radius=50 mask=0.7"); // correction for blurred image
        run("Threshold...");
```

```
	waitForUser("Pause","Set threshold");
	setOption("BlackBackground", true);
	run("Convert to Mask");
	run("Make Binary");
	run("Watershed");
	saveAs("Tiff", output+title);

	run("Analyze Particles...", "size=50-500 show=[Overlay Masks] summarize overlay");
	run("Flatten");
	saveAs("Jpeg", output2+title);
	close();
}

//----------------------------------------------------------------------------------------------------------------
//Function for nuclei skeletonization for directionality analysis
//----------------------------------------------------------------------------------------------------------------
function processFile(inputFolder, output, file) {
	open(inputFolder + file);
	title = getTitle();
	print(title);

	run("Skeletonize");
	run("Maximum...", "radius=2");
	run("Skeletonize");
	saveAs("Tiff", output + title); //save segmented imagetitle = getTitle();
	close();
}

//----------------------------------------------------------------------------------------------------------------
//Function for intervessel network analysis of WEKA-segmented images
//----------------------------------------------------------------------------------------------------------------
function processFile(inputFolder, output, file) {
	open(inputFolder + file);
	title = getTitle();
	print(title);

	run("Skeletonize");
	saveAs("tiff", output + "Skeleton " + title);
	run("Analyze Skeleton (2D/3D)", "prune=none show");

	selectWindow("Results");
	DetResults = getInfo("window.contents");

	for(i=0; i<nResults; i++) {
	totBranchNumber += getResult("# Branches", i);
	totJunctionNumber += getResult("# Junctions", i);
	totLength += getResult("Branch length", i);
	}
	close("Results");

	selectWindow("Tagged skeleton");
	saveAs("tiff", output + "Tagged " + title);
	close();
```

```
        print("TotalBranchNumber: " + totBranchNumber);
        print("TotalJunctionNumber: " + totJunctionNumber);
        print("TotalBranchLength: " + totLength);
}
```
